# Supplementary material for: Highly local activation of inhibition at the seizure wavefront in vivo
Source: Cell Rep. 2024 May 3;43(5):114189. doi: 10.1016/j.celrep.2024.114189 (PMC11913739; doi:10.1016/j.celrep.2024.114189)
Supplement: Document S1. Figures S1–S7 [file mmc1.pdf]

Cell Reports, Volume *43*

Supplemental information

Highly local activation of inhibition at the seizure wavefront *in vivo*

Prajay T. Shah, Taufik A. Valiante, and Adam M. Packer

Supplemental Information

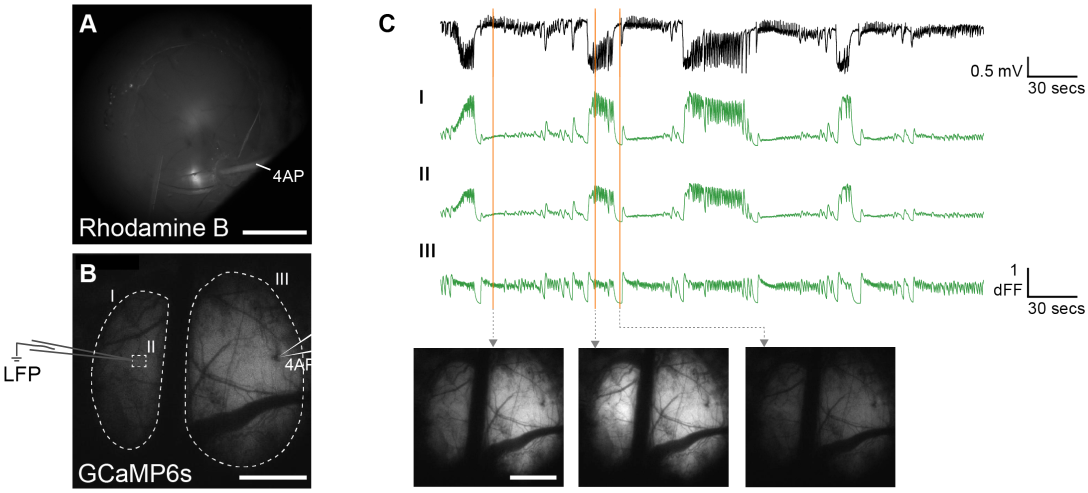

Figure S1. Cross-hemispheric, widefield calcium imaging of focal onset seizure. Related to Figure 1.

A. Focal 4-AP injection (mixed with Rhodamine-B) near the edge of the implanted cranial window. Note the fluorescence signal at the centre of the cranial window in this imaging channel is from viral-injection-transduced expression of C1V1. Scale bar = 1 mm

B. Widefield, cross-hemispheric calcium imaging under a craniotomy over the frontal cortex. LFP signal was collected contralateral to the focal 4-AP injection. ROIs were selected for fluorescence signal collection relative to the 4-AP injection and LFP recording electrode (I, II and III). Scale bar = 1 mm

C. Average fluorescence signal (green traces) from various ROIs marked in panel B, synchronised to the simultaneously collected LFP trace (black trace). Below is shown single-frame widefield calcium images during interictal, ictal and following seizure termination. Orange lines represent time points of imaging frames shown below. Scale bar = 1 mm

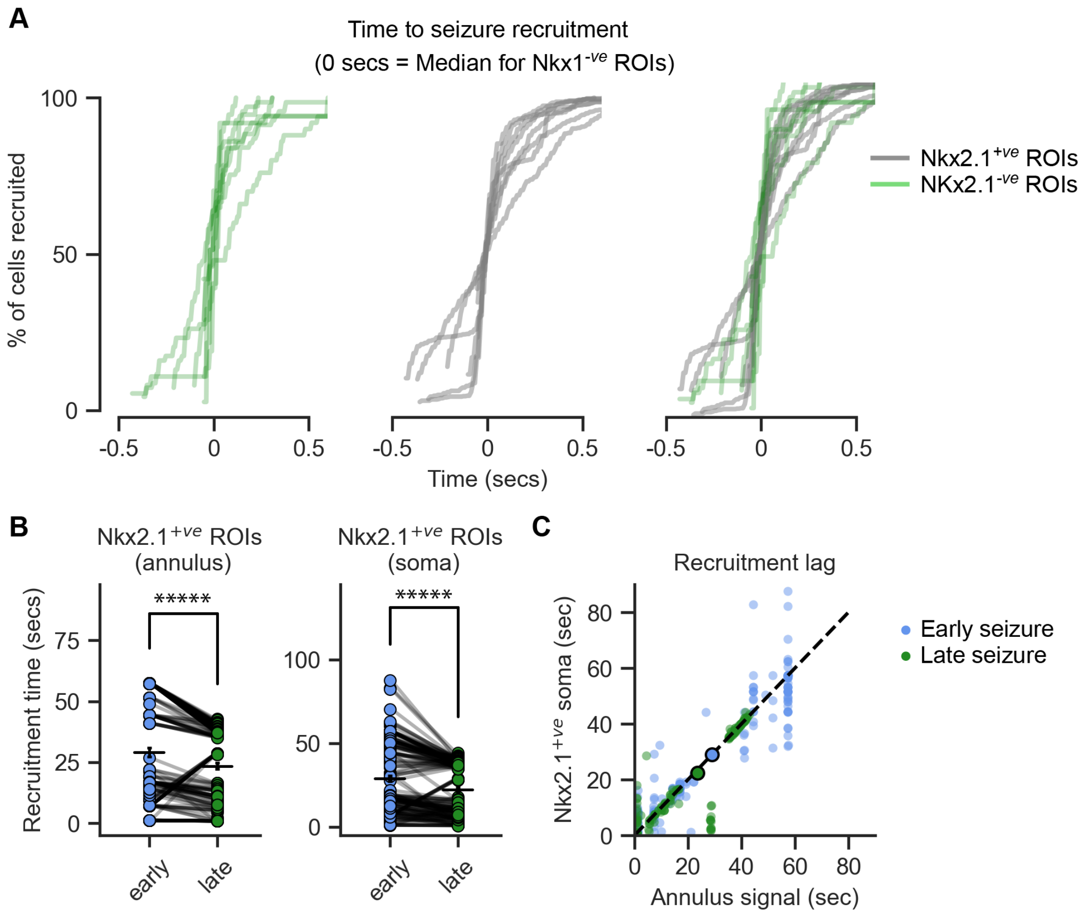

Figure S2. Additional quantification of inhibitory neuron recruitment during seizure propagation. Related to Figure 1.

A. Cumulative time to recruitment of Nkx2.1-expressing inhibitory neurons and Nkx2.1-negative putative-excitatory neurons of all analyzed seizures. Time to recruitment of both Nkx2.1-positive and Nkx2.1-negative cells is normalized to the maximum time to recruitment of Nkx2.1-negative cells of each seizure. Time = 0 sec represents the median time to recruitment of Nkx2.1-negative cells of each seizure.

B. Seizure recruitment time for the annulus (left) and soma (right) of Nkx-2.1-positive ROIs, divided by early and late seizures (from n = 3 mice). The seizure recruitment time is modestly decreased for later seizures for both soma and annulus signals. *****$p<10e-5$, paired t-test (individual ROIs are compared early vs. late seizures).

C. The recruitment lag of each Nkx2.1-positive ROI's annulus and soma signals, grouped by early (blue) and late (green) seizures. The larger points with black outline represent the mean for each group.

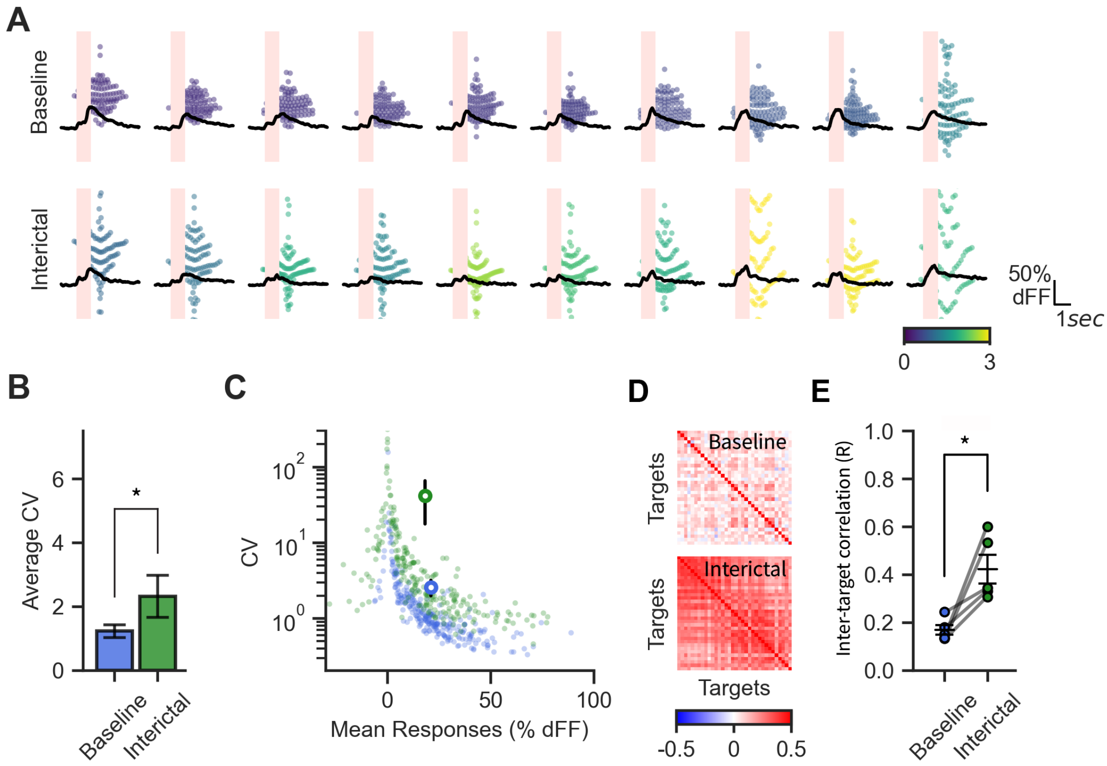

Figure S3. Dynamic variability of neuronal excitability across brain states. Related to Figure 3.

A. The coefficient of variation (CV) measured trial-by-trial variability in photostimulation response magnitude of individual photostimulation targeted neurons. Representative examples of 10 randomly selected individual targets (matched Baseline (top) and Interictal (bottom) states) from a single experiment sorted by increasing CV; black trace is the mean photostimulation timed response, scatter dots are individual photostimulation responses colored by the CV of responses of each respective neuron.

B. Average |coefficient of variation| (CV) of photostimulation targets under baseline and interictal conditions (N = 6 mice; *MWU: $p < 0.05$).

C. Relationship of average photostimulation response magnitude and CV of photostimulation targets across all experiments (2-way ANOVA: p(Binned responses, 20 µm bins) = 3.8e-39, p(Group:Binned responses) = 8.34e-18). Open circles represent mean locations for Baseline (blue circle) and Interictal (green circle) points (errobars: +/- SEM). Excluded datapoints: CV > 300, Mean Responses (% dFF) < -30.

D. Inter-neuronal cross-correlation matrix across all photostimulation targets' z-scored responses to all trials from one representative experiment (left: Baseline, right: Interictal).

E. Increased inter-neuronal correlation of photostimulation targets during interictal period (N=5, paired t-test: *p(baseline vs. interictal)<0.05).

CV: Absolute value of coefficient of variation. Error bars: Mean +/- SEM

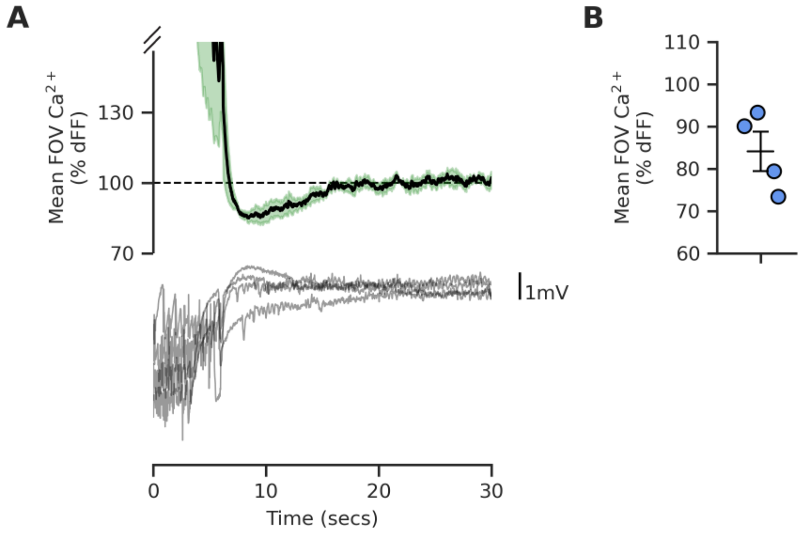

Figure S4. Ca^{2+} signal after seizure termination. Related to Figure 3.

A. Dynamics of the raw FOV Ca^{2+} signal in the post-seizure termination period from a representative experiment (mean +/- SEM; normalised to the interictal period of same experiment).

B. Mean (+/- SEM) of post-seizure termination normalised Ca^{2+} signal across experiments.

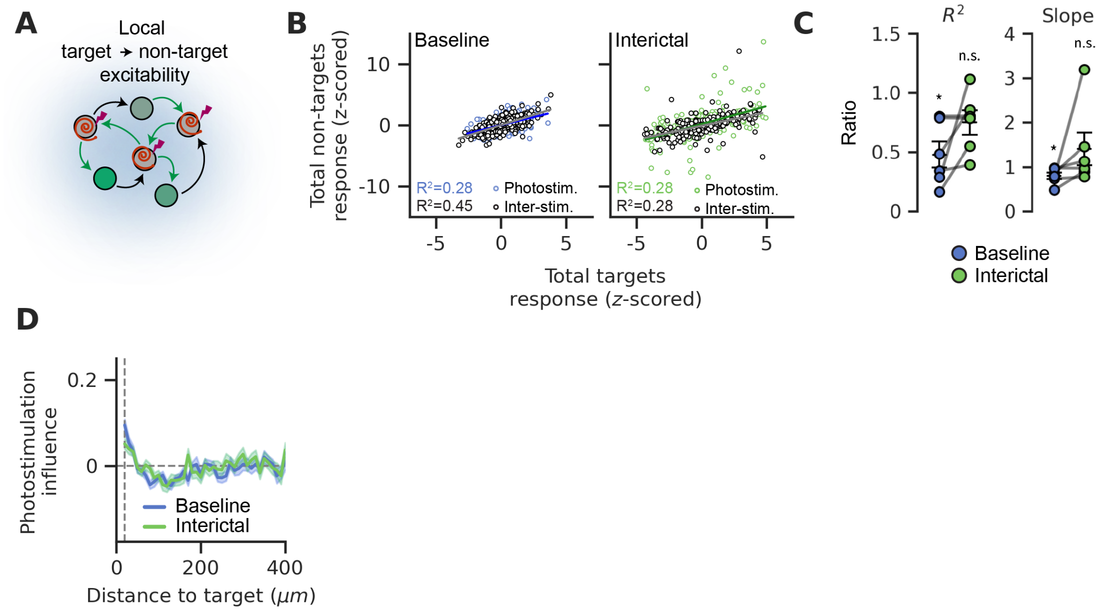

Figure S5. Local circuit excitability in baseline and interictal states. Related to Figure 3.

A. Local circuit excitability is analyzed as the interaction between photostimulation of target neurons and the responses of non-target neurons within the entire FOV of each experiment. The response of each non-target neuron was compared to the response of the closest photostimulation target by Euclidean distance was measured.

B. Relationship of single-trial total response of targeted neurons vs. total response of non-targeted neurons in baseline (left, blue) and interictal (right, green) states. Activity levels of each trial were z-scored within each experiment to allow for comparison across experiments. Inter-stimulation (artificial) trials (open circles) were defined between photostimulation trials to serve as control comparison within each experiment. Each point is a stimulation trial, all datapoints across all experiments are shown (N=6 mice, n = 530 baseline trials and 309 interictal trials, Pearson's R total response targets vs. non targets: p(baseline, photostimulation) = 2.1e-39, p(baseline, inter-stimulation) = 1e-70; p(interictal, photostimulation) = 7.0e-24, p(interictal, inter-stimulation) = 2e-22).

C. R2 and linear regression slope of photostimulation trials vs. inter-stimulation (artificial) trials for baseline (blue) and interictal (green) states (N = 6 mice, Wilcoxon signed-rank test, two-tailed comparison for different than 1, *$p < 0.05$). The ratio of photostimulation trials to inter-stimulation (artificial) trials is shown to demonstrate that during the baseline state, photostimulation results in lower non-target responses compared to inter-stimulation trials. However, this is not the case in the interictal state, suggesting an increase of local circuit excitability in this state.

D. The influence of photostimulation on the activity of non-target neurons (n = 8567) relative to their distance (10 μm bins) from the nearest target neuron un-

der baseline (blue) and interictal (green) states (2-way ANOVA: p(Distance) = 5.5e-51, p(Group) = 0.80, and p(Group:Distance) = 0.35).

n.s.: not significant. Error bars and spans: Mean +/- SEM.

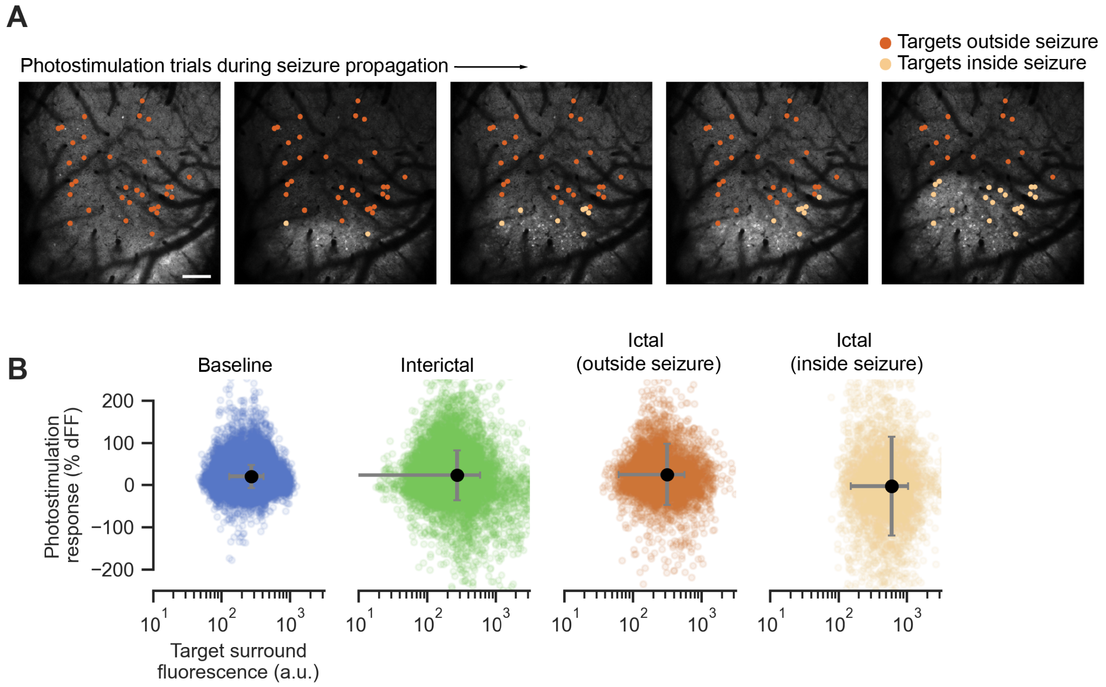

Figure S6: Seizure boundary classification. Related to Figure 4.

A. Calcium fluorescence images of consecutive photostimulation trials during seizure propagation of a single representative ictal event from one experiment. Fluorescence images are 3 secs average around time of photostimulation trial. All photostimulation targets in each experiment were individually classified as being inside or outside the seizure wavefront boundary on a given photostimulation trial during ictal events. Photostimulation trials with unclear seizure wavefront locations (i.e. wavefront has not yet reached FOV, or wavefront has passed FOV) did not receive this classification for targets' location. Scale bar = 200 µm

B. Relationship of surrounding fluorescence (raw) signal to photostimulation response of all targets and photostimulation trials across all experiments. Ictal photostimulation trials were further split by individual targets' location inside/outside the seizure wavefront boundary at each individual photostimulation trial.

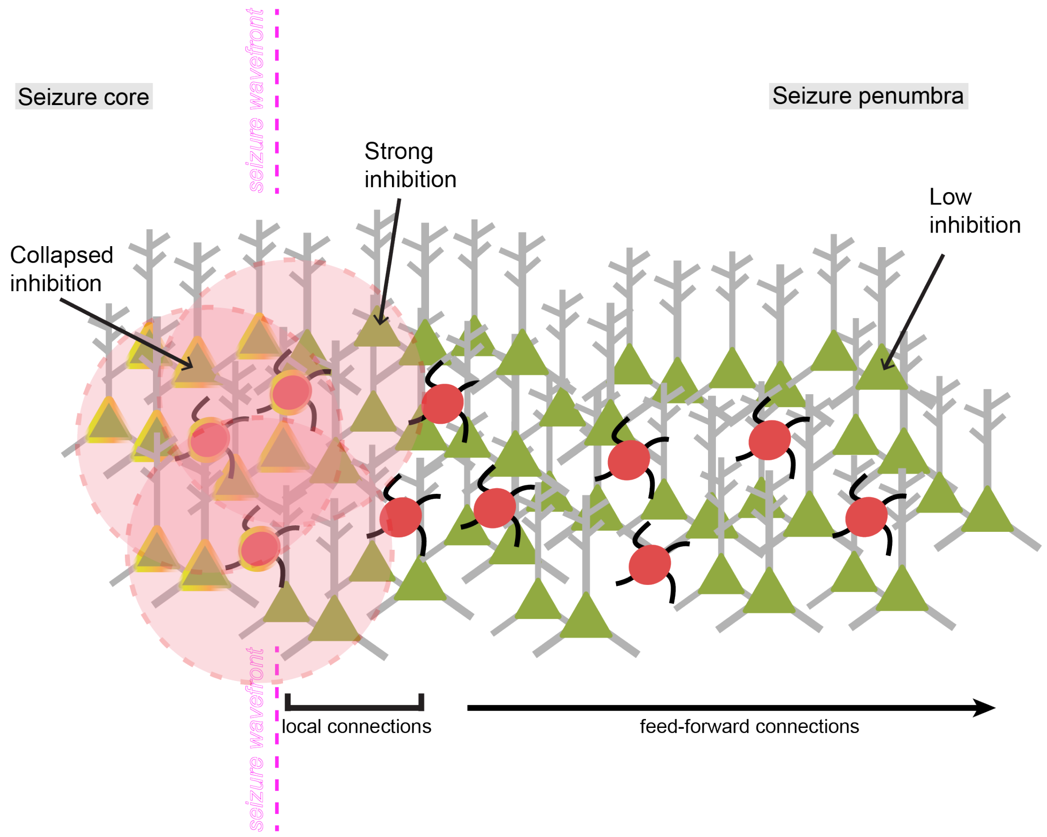

Figure S7: The proposed model of inhibition relative to the seizure wavefront. Related to Figure 4.

The boundary of an actively propagating seizure wavefront includes intense activation of both excitatory (green triangles) and inhibitory neurons (red circles). The intense activation of inhibitory neurons at the edge of the seizure boundary creates a region of strong inhibition in region directly proximal to the seizure wavefront. This occurs in a profile that follows the highly-local anatomical connectivity of cortical interneurons. In contrast, inhibitory neurons in the distal seizure penumbra are not activated ahead of the ictal wavefront, creating a low inhibition state in the distal seizure penumbra.
