# Supplementary material for: Highly local activation of inhibition at the seizure wavefront in vivo
Source: Cell Rep. 2024 May 3;43(5):114189. doi: 10.1016/j.celrep.2024.114189 (PMC11913739; doi:10.1016/j.celrep.2024.114189)
Supplement: Document S2. Article plus supplemental information [file mmc2.pdf]

Cell Reports

Report

Highly local activation of inhibition at the seizure wavefront *in vivo*

Graphical abstract

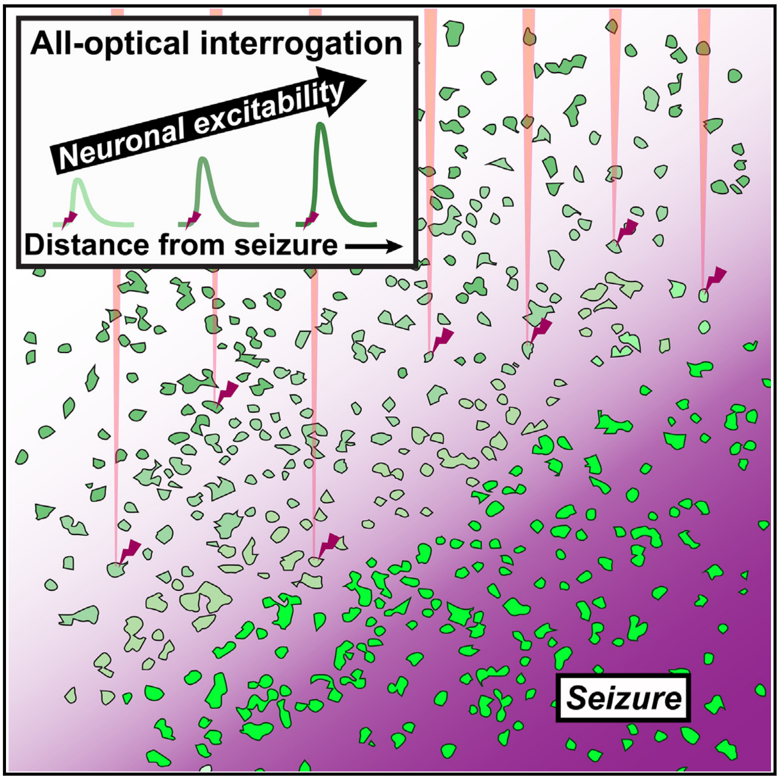

Highlights

- Inhibitory neurons are not activated distal to the seizure wavefront
- All-optical interrogation measures neuronal excitability in acute focal-onset seizure
- Single-neuron excitability is stable during interictal periods
- Single-neuron excitability is decreased only near the seizure wavefront

Authors

Prajay T. Shah, Taufik A. Valiante, Adam M. Packer

Correspondence

adampacker@gmail.com

In brief

Shah et al. describe an approach to measure single-neuronal excitability at large scale in a mouse model of epilepsy *in vivo* and show that neuronal excitability is reduced near a seizure wavefront while remaining preserved throughout the greater seizure penumbra.

Shah et al., 2024, Cell Reports *43*, 114189
May 28, 2024 © 2024 The Authors. Published by Elsevier Inc.
https://doi.org/10.1016/j.celrep.2024.114189

CellPress

Cell Reports

CellPress
OPEN ACCESS

Report

Highly local activation of inhibition at the seizure wavefront *in vivo*

Prajay T. Shah,[1,2] Taufik A. Valiante,[1,2,3,4,5] and Adam M. Packer[6,7,*]
[1]Krembil Brain Institute, University Health Network, Toronto, ON, Canada
[2]Institute of Biomedical Engineering, University of Toronto, Toronto, ON, Canada
[3]Department of Electrical and Computer Engineering, University of Toronto, Toronto, ON, Canada
[4]Institute of Medical Sciences, University of Toronto, Toronto, ON, Canada
[5]Division of Neurosurgery, Department of Surgery, University of Toronto, Toronto, ON, Canada
[6]Department of Physiology, Anatomy, and Genetics, University of Oxford, Oxford, UK
[7]Lead contact
*Correspondence: adampacker@gmail.com
https://doi.org/10.1016/j.celrep.2024.114189

SUMMARY

The propagation of a seizure wavefront in the cortex divides an intensely firing seizure core from a low-firing seizure penumbra. Seizure propagation is currently thought to generate strong activation of inhibition in the seizure penumbra that leads to its decreased neuronal firing. However, the direct measurement of neuronal excitability during seizures has been difficult to perform *in vivo*. We used simultaneous optogenetics and calcium imaging (all-optical interrogation) to characterize real-time neuronal excitability in an acute mouse model of seizure propagation. We find that single-neuron excitability is decreased in close proximity to the seizure wavefront but becomes increased distal to the seizure wavefront. This suggests that inhibitory neurons of the seizure wavefront create a proximal circumference of hypoexcitability but do not influence neuronal excitability in the penumbra.

INTRODUCTION

The activity of neurons is determined by subthreshold dynamics influencing neuronal excitability. The regulation of neuronal excitability is normally achieved by a tight circuit-level balance of excitatory and inhibitory inputs,[1] but this fails in disordered states like seizures wherein large-scale neuronal populations gain massive hyperexcitability. In focal-onset seizures, a spatiotemporally propagating seizure wavefront divides the intensely active seizure core and the hypoactive seizure penumbra.[2,3] Whereas the hyperexcitability of the seizure core is easily explained by inhibitory collapse, the seizure penumbra is suggested to be characterized by heightened inhibition (so-called "surround inhibition").[2,4–12]

There is evidence of large GABAergic activity ahead of seizure recruitment in whole-cell electrophysiological recordings *in vitro*, but the spatial extent of this inhibition and its applicability to seizures *in vivo* remain unclear. Although live imaging studies of inhibitory neurons during seizure propagation *in vivo* have also been performed,[5,13] these lacked simultaneous measurement and/or analysis of (1) the seizure wavefront, (2) the activity of excitatory neurons, and (3) the activity of inhibitory neurons. Ultimately, these studies do not sufficiently answer whether post-synaptic excitability of neurons in the penumbra is decreased as per the circuit-level mechanisms proposed in the surround inhibition model.

Measuring the effect of seizure-related circuit mechanisms on neuronal excitability requires methods of direct perturbations of neurons *in vivo*. All-optical interrogation combines calcium imaging with targeted optogenetics to provide a method for measuring neuron excitability at single-cell resolution in correlation with real-time seizure propagation *in vivo*. Here, we set out to characterize the profile of inhibition *in vivo* using cell-type-specific calcium imaging and direct probing of neuronal excitability in a mouse model of focal seizure propagation.

RESULTS

To visualize real-time seizure propagation, we performed awake, head-fixed calcium imaging and local field potential (LFP) recording combined with a focal injection of 4-aminopyridine (4-AP) in the cortex of mice (Figures 1A and 1B). The focal 4-AP model is characterized by brief, spontaneously occurring seizures (ictal periods) with high neuronal spiking and interictal periods of quiescence (Figures 1D and 1E). Cross-hemispheric widefield calcium imaging shows locally increased activity at the site of 4-AP injection (Figure S1), but this does not lead to increased activity in other locations until the seizure has recruited those regions. Two-photon (2P) calcium imaging distal to the 4-AP injection site demonstrated seizure propagation as a gradual recruitment of neurons across the experimental field of view (FOV; Figure 1C).

Focal-seizure onset is thought to be associated with a fast rise in the activity of inhibitory neurons in the penumbra.[5,13,14] However, previous studies were limited by a small sample size of

This is an open access article under the CC BY license (http://creativecommons.org/licenses/by/4.0/).

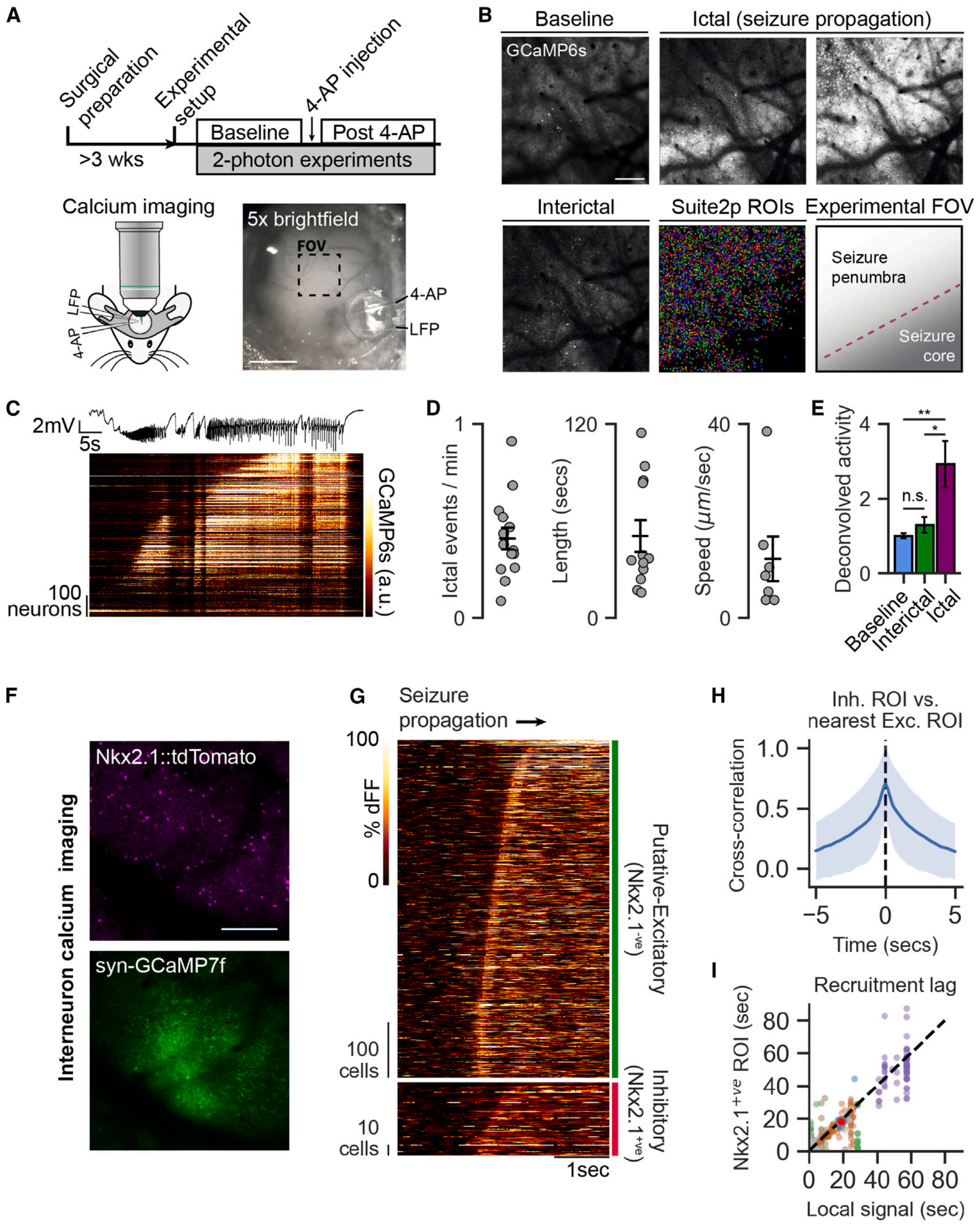

Figure 1. Similar activation of excitatory and inhibitory neurons during seizure propagation

(A) Experimental workflow (top) and setup of simultaneous *in vivo* 2P imaging, local field potential (LFP) recording, and 4-aminopyridine (4-AP) injection in awake, head-fixed mice over somatosensory cortex. Calcium imaging was performed in CaMKIIa-expressing excitatory neurons transgenically or virally induced to express GCaMP6s and virally induced C1V1-Kv2.1-mScarlet for subsequent optogenetic experiments. 4-AP was injected near the site of LFP recording and approximately >1 mm away from the experimental microscope FOV. Bottom right: bright-field image of cranial window showing locations of imaging FOV, 4-AP injection pipette, and LFP-recording pipette during a representative experimental session. Scale bar: 1 mm.

(B) Imaging experiments were performed at the same microscope FOV during baseline (pre-4-AP injection), interictal (post-4-AP injection, no seizure), and ictal (post-4-AP injection, during seizure). Shown are multi-frame average projections of a representative calcium imaging time series during an experiment. (Bottom middle) Cell masks collected from Suite2p processing of baseline calcium imaging. (Bottom right) During seizure propagation, the seizure wavefront divides the seizure core from the seizure penumbra. Scale bar: 200 μm.

(legend continued on next page)

inhibitory neurons, and the activity dynamics of excitatory neurons was not simultaneously measured, thus it remains unclear whether the increased activity of inhibitory neurons leads to inhibited excitatory activity in the penumbra. We performed cell-type-specific calcium imaging using viral injection of pan-neuronal GCaMP7f in Nkx2.1-Cre::mCherry mice (Figure 1F), which have been reported to label ~60% of PV- and SST-expressing neurons in L2–4 of mouse cortex.[15,16] This preparation yielded imaging of 52 ± 13 (mean ± SD) Nkx2.1^{+ve} inhibitory neurons from each animal (*N* = 3 animals) along with their surrounding Nkx2.1^{-ve} (putatively-excitatory) neurons.

Although calcium imaging does not afford temporal resolution comparable to previous whole-cell electrophysiological studies of inhibitory neurons during seizure propagation, we gain a key benefit of greater spatial resolution to closely relate the spatial activity dynamics of inhibitory neurons, excitatory neurons, and the seizure wavefront itself. We found that following seizure onset, the recruitment of Nkx2.1^{+ve} inhibitory neurons was overall closely associated with that of Nkx2.1^{-ve} neurons (Figures 1G and S2). There was high cross-correlation of the calcium signal of each inhibitory neuron and its nearest excitatory neuron (Figure 1H). To measure the timescale of Nkx2.1^{+ve} neuron activation relative to seizure propagation, we analyzed the recruitment of Nkx2.1^{+ve} neurons against the average signal of a 200-μm-diameter annulus centered on each Nkx2.1^{+ve} neuron (excluding other Nkx2.1^{+ve} neurons within the annulus). This demonstrated that the recruitment of Nkx2.1^{+ve} neurons was not significantly different from the propagation of the seizure wavefront into their local region (Figure 1I). This also did not evolve between earlier and later seizures relative to 4-AP injection (Figure S2). Thus, we did not find any evidence for differences in time of recruitment between inhibitory neurons and local excitatory neurons during seizure propagation.

Our calcium imaging of inhibitory neurons showed they are activated in concert with the leading edge of the seizure wavefront, but how does this manifest in neuronal excitability? To study how neuronal excitability is affected in our acute epilepsy model and during seizure propagation, we performed simultaneous optogenetics and calcium imaging. The somatically targeted opsin C1V1-Kv2.1 and GCaMP6s were co-expressed distal to the 4-AP injection/LFP recording site (Figure 2A). We first characterized the network-scale excitability using widefield optogenetic photostimulation, which evoked a robust and reliable response in the mean FOV calcium imaging and LFP signals (Figure 2B). Given the spatial separation between C1V1-expressing neurons at the center of the cranial window and the LFP recording site at the cranial window edge, the LFP response represents optogenetically evoked activity propagating to the 4-AP/LFP site. Indeed, 78% of ictal onsets in these experiments were correlated to photostimulation trials (Figure 2C, *p*(V test for non-uniformity) = 1.3e−4, time bin = −500 to +500 ms relative to photostimulation), suggesting that many seizures were directly induced by photostimulation. During the interictal periods, there was a significant increase in the photostimulation-evoked calcium response magnitude and the decay constant of the post-stimulation calcium trace (Figures 2D–2F; mean ± SD; response magnitude: 0.62 delta F/F [dFF] ± 0.66, decay constant: 0.74 s ± 0.40; t test [response magnitude, baseline vs. interictal]: *p* = 5e−25, t test [decay constants, baseline vs. interictal]: *p* = 6e−18; trials within 500 ms of ictal onset and offset were excluded). This increased population GCaMP response magnitude and decay constant of the photostimulated population of neurons demonstrate a widescale hyperexcitability and sensitivity to seizure established by the focal 4-AP model.

To distinguish if single-neuronal excitability is affected distal to the 4-AP site, we used 2P holographic photostimulation (all-optical interrogation) of 30–50 selected neurons in each experimental FOV (Figure 3A, *N* = 6 mice, 41.7 ± 9.5 [mean ± SD] targets per animal). Under the baseline state, photostimulation evoked a strong mean calcium response from targeted neurons across repeated trials (mean ± SD: 21.3% dFF ± 9.3). Unlike with widefield stimulation, there was no correlation with seizure incidence (Figures 3B and 3C). Moreover, during interictal periods (Figures 3D and 3E), there was no significant difference in the average photostimulation response across targeted neurons (% dFF mean ± SD: 17.4 ± 13.1, paired t test [baseline vs. interictal]: *p* = 0.22). This is consistent with the comparable average pre-photostimulation GCaMP-fluorescence levels for photostimulation-targeted neurons (Figure 3F, paired t test *p*(baseline vs. interictal) = 0.85). We also measured the positively correlated

(C) Calcium imaging signal of neurons from imaging FOV (bottom, heatmap) and corresponding LFP (top, black trace) during one representative seizure.
(D) Average frequency of seizure incidence (left), average duration of seizures (middle), and average propagation speed of seizure (right) after 4-AP injection measured from calcium imaging experiments.
(E) Neuronal activity across baseline, interictal, and ictal periods (mean ± SEM). Activity rate for each neuron was calculated as the area underneath the curve of Suite2p's deconvolved spike output signal divided by the total length of recording. One-way ANOVA and Tukey post-hoc comparison: *p*(baseline vs. interictal) > 0.05, ***p*(baseline vs. ictal) < 0.01, and **p*(interictal vs. ictal) < 0.05 (*N* = 6 mice).
(F) Nkx2.1-Cre-tdTomato mice were virally transfected with syn-GCaMP7f for calcium imaging of Nkx2.1-expressing inhibitory neurons and non-Nkx2.1-expressing (putatively-excitatory) neurons. (Top) Inhibitory neurons labeled with tdTomato (780 nm excitation/red emission multi-frame average). (Bottom) Pan-neuronal expression of GCaMP7f (920 nm excitation/green emission multi-frame average). Scale bar: 100 μm.
(G) Cell-type-specific GCaMP7f fluorescence signal (dFF, delta F/F) at −1.5 to +2.5 s around seizure onset in a representative seizure event. Fluorescence signal of Nkx2.1^{-ve} (putatively-excitatory, green bar) and Nkx2.1^{+ve} (inhibitory, red bar) neurons. Neurons are ordered based on delay to seizure recruitment (measured as reaching 85% of their maximal signal). Signal is normalized to the 1.5–0.5 s period prior to seizure onset.
(H) Cross-correlation lag between each inhibitory neuron and the nearest excitatory neuron (within 100 μm) from 1 s prior to seizure onset and 1 s following seizure termination (shown is average across all inhibitory neuron and all seizures, *n* = 10 seizures from *N* = 3 mice).
(I) Delay to seizure recruitment of inhibitory neurons compared to their local neuropil signal (measured as average signal from 100 μm annulus around each ROI). Each individual point represents a single inhibitory neuronal ROI, and each color represents a single seizure event. Red point represents the average of all points. Dashed line is x = y line (*n* = 10 seizures from *N* = 3 mice; n.s. *p*(paired t test) > 0.05).
Error bars and spans: mean ± SEM.
See also Figures S1 and S2.

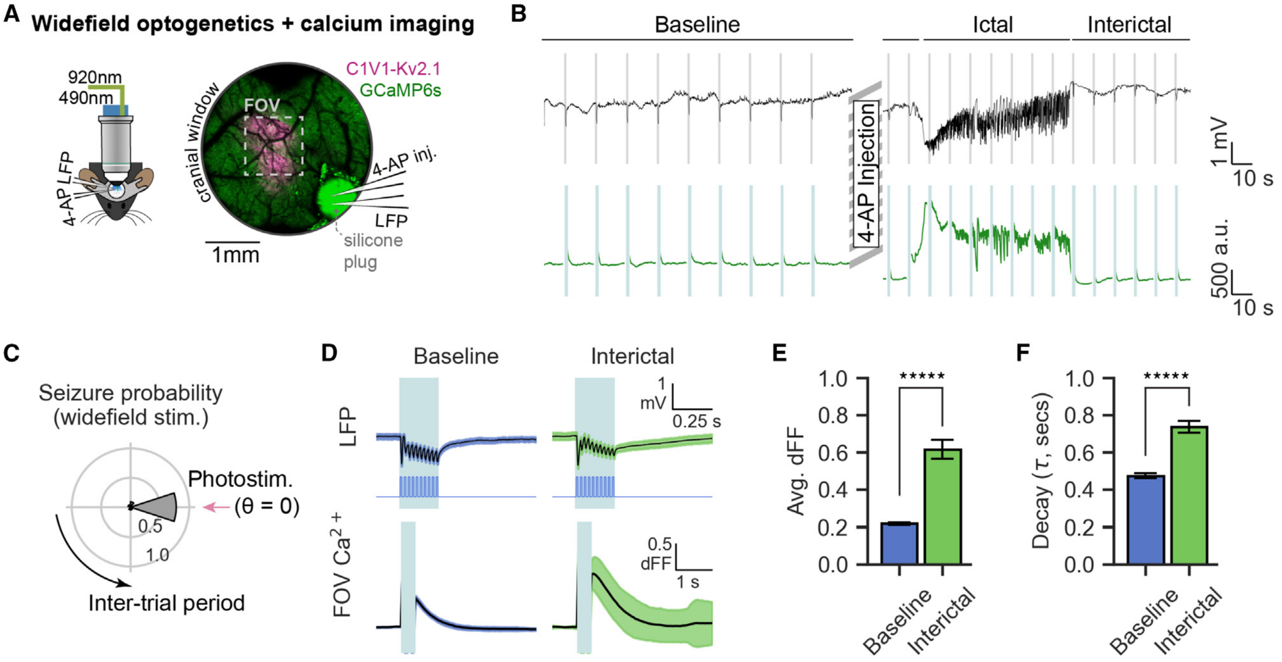

Figure 2. Increased network excitability during the interictal period

(A) (Left) Experimental setup for combined head-fixed 2P calcium imaging and widefield (1P) optogenetic photostimulation with LFP recording and 4-AP injection. (Right) 2P image of representative cranial window showing viral co-expression of GCaMP6s (green) and C1V1-Kv2.1-mScarlet (magenta). 4-AP and LFP pipettes are inserted through the access hole in the cranial window after removal of the silicone plug.

(B) LFP recording (top) and corresponding mean FOV fluorescence calcium imaging signal (bottom) from a single representative experiment before and after 4-AP injection. Blue stripes represent widefield photostimulation trials. High-speed shutters covering the imaging detectors were triggered for protection during widefield illumination.

(C) Probability of seizure occurrence relative to photostimulation timing across all seizure events (1 s bins, $N = 5$ mice, p(V test for circular non-uniformity) = 1.3e−4).

(D) Photostimulation-timed average response (±SD) of LFP signal and widefield illumination TTL trigger output (top) and FOV-average calcium imaging signal (bottom) from a representative experiment. Blue span represents widefield illumination.

(E) Average photostimulation response magnitude during baseline and interictal states (N = 5–6 mice per group, 360 baseline trials and 172 interictal trials [trials inducing seizures were excluded]; t test: *****$p <$ 1e−24).

(F) Average decay constant of photostimulation response across baseline and interictal states (N = 5–6 mice per group, 359 baseline trials and 156 interictal trials [trials inducing seizures were excluded], t test: *****$p <$ 1e−17). Decay constant calculated as the time (s) post-stimulation when the signal decreases below 63% of the maximum post-stimulation value.

Error bars and spans: mean ± SEM.

trial-by-trial dynamic variability of single-neuron excitability, which became significantly more correlated in the interictal period (Figure S3). Thus, in the interictal period, the average single-neuron excitability of neurons distal to the seizure-onset zone remains comparable to baseline levels but is more correlated between neurons.

Is there a change in distal neuronal excitability before seizure onset or after seizure termination? Particularly, the post-ictal period displays profound, brain-wide suppression in electroencephalography,[17,18] as well as in calcium imaging (Figure S4). Interestingly, there was no change in neuronal excitability within 30 s prior to seizure onset, yet there was an increase in neuronal excitability during the 30 s after seizure termination (Figure 3G).

All-optical interrogation can also be used to map the influence of photostimulated neurons to non-target neurons.[19–26] A modified influence metric (see STAR Methods) was used to measure the average influence of photostimulation of a given target neuron on non-targeted neurons within 400 μm. As previously reported,[20,21,26,27] the influence of photostimulation was excitatory for nearby neurons and inhibitory for neurons up to ∼200 μm away (Figure S5). This influence profile with respect to distance was not significantly altered during the interictal state, and there was no significant difference in the influence measure between baseline and interictal (Figure S5, two-way ANOVA; distance to target: $p <$ 1e−27; trial state: $p >$ 0.05).

Finally, how is the excitability of neurons in the penumbra affected during seizure propagation? The location of the seizure wavefront was identified in all photostimulation trials that took place during an LFP-signal-marked ictal event (Figure S6). Single-trial responses of photostimulated neurons were Z scored to baseline in a target-wise manner and compared to the seizure wavefront (Figure 4A). There was a significant relationship of the photostimulation responses of targeted neurons in relation to their distance from the seizure wavefront (Figure 4B, one-way ANOVA: p = 1.2e−12; 64,024 total measurements, n = 154 stimulation trials with seizure

CellPress
OPEN ACCESS

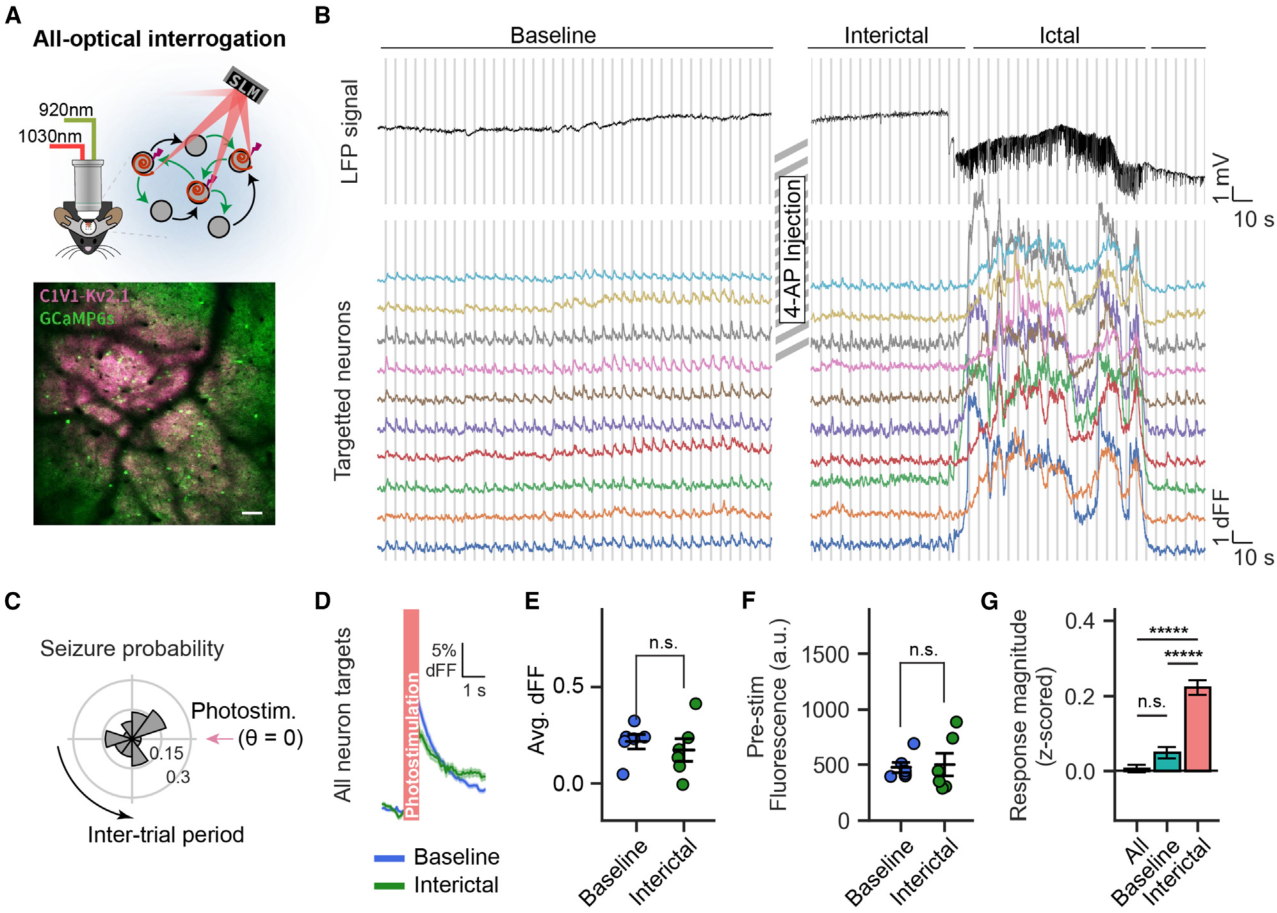

Figure 3. Single-neuronal excitability is not increased during the interictal period

(A) (Top) Head-fixed 2P all-optical interrogation. (Bottom) Co-expression of GCaMP6s (green) and C1V1-Kv2.1-mScarlet (pink) at the experimental microscope FOV. Scale bar: 100 μm.

(B) All-optical interrogation performed at a fixed FOV over three states: baseline (pre-4-AP injection), interictal (post-4-AP injection, non-seizure), and ictal (post-4-AP injection, during seizure). (Top) LFP recording from a single experiment before and after 4-AP injection. (Bottom) Calcium fluorescence traces from photostimulation targeted neurons during baseline and the same targets during an ictal event following 4-AP injection. Photostimulation trials are represented by vertical lines over the LFP and calcium fluorescence traces.

(C) The probability of seizure occurrence relative to photostimulation timing (binned at 1 s intervals, $N = 6$ mice, p(V test for circular non-uniformity) = 0.47).

(D) Photostimulation timed dFF normalized fluorescence response of photostimulation targeted neurons under baseline and interictal states (shown as mean ± SEM across all targets in all experiments).

(E) Photostimulation evoked response magnitude of photostimulation targets in each experiment ($N = 6$ mice, paired t test: $p = 0.22$).

(F) FOV fluorescence of photostimulation targets measured from a 500 ms period before each photostimulation trial ($N = 6$ mice; paired t test: $p = 0.85$).

(G) Average photostimulation responses of all targeted neurons and interictal photostimulation trials (calculated as Z scores of dFF responses relative to baseline) classified into subperiods: all (all interictal trials), pre-ictal (within 30 s prior to seizure onset), and post-ictal (within 30 s after seizure termination). One-way ANOVA and Tukey post-hoc: *****p(interictal vs. post-ictal) < 1.7e−13, *****p(pre-ictal vs. post-ictal) < 1.8e−13), n.s. p(interictal vs. pre-ictal) = 0.12).

mV, millivolts; n.s., not significant. Error bars and spans: mean ± SEM.

See also Figures S3–S5.

wavefronts, $N = 6$ experiments). Specifically, photostimulation responses of targeted neurons are suppressed in close proximity to the seizure wavefront (<~100 μm) while being increased distal to the seizure wavefront (> ~100 μm) relative to their baseline responses. There was also a significant relationship of the neuropil signal of neuronal regions of interest to their distance from the seizure wavefront (Figure 4B, one-way ANOVA: $p < 10e{-}5$). Conversely, photostimulation during seizure propagation evoked greater responses in proximal non-targeted neurons (<100 μm to the seizure wavefront) compared to non-targeted neurons distal (>200 μm to the seizure wavefront) (Figure 4C). The relationship of photostimulation influence to anatomical space between target and non-target neurons was not present proximal to the seizure wavefront (Figure 4D, one-way ANOVA: $p = 0.17$) but was still present in the distal zone (Figure 4D, one-way ANOVA: $p = 0.02$). These heightened responses of non-targets, in contrast to the decreased responses of targets, may result from differences between photostimulation-driven (i.e., soma-targeted) vs. propagated excitation. In conclusion, by using an approach

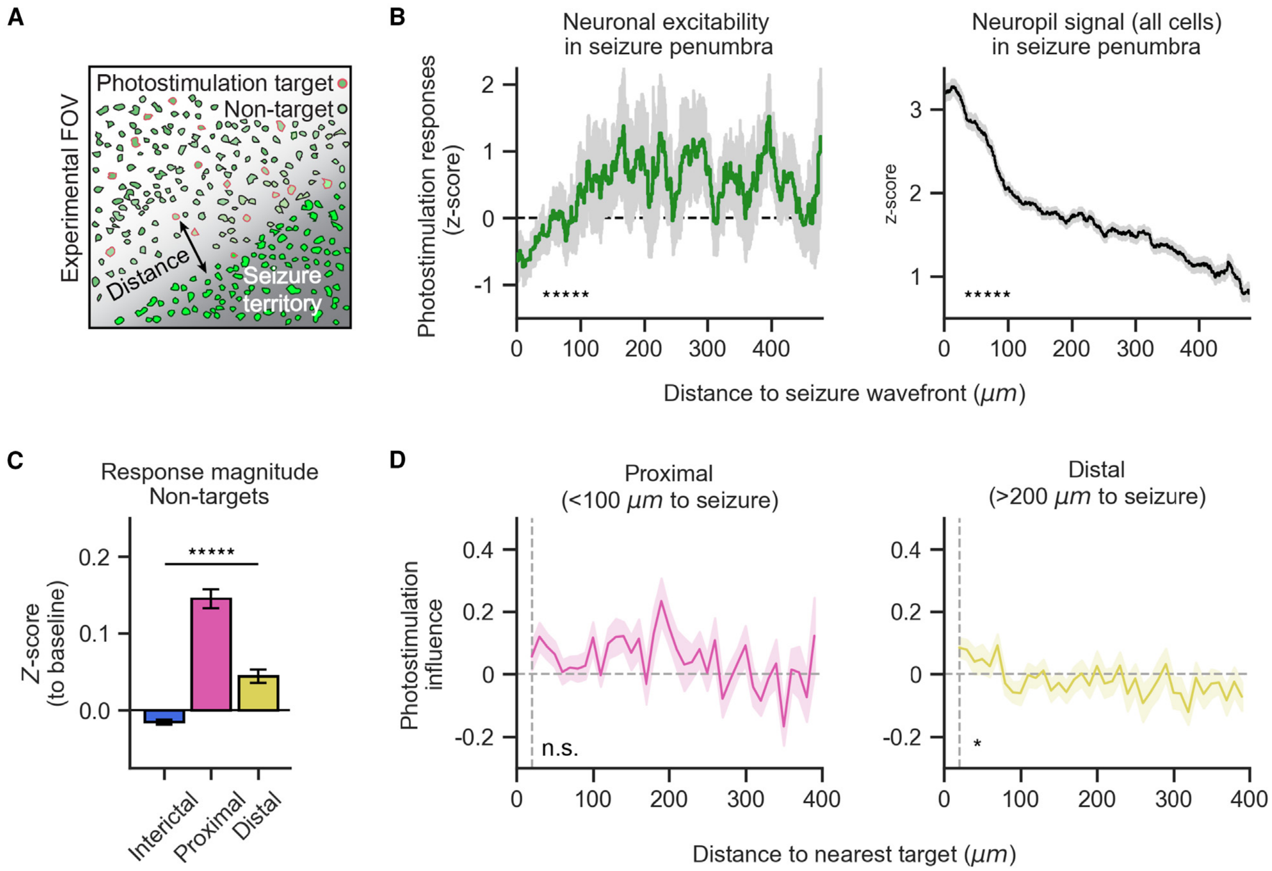

Figure 4. Decreased single-neuronal excitability near the seizure wavefront

(A) Distance to seizure wavefront (dashed line) was measured linearly for photostimulation target cells (red + green filled) and non-targeted cells (green filled only) at each photostimulation trial during seizure spread inside the FOV.

(B) (Left) Profile of photostimulation responses of targeted neurons over distance (40 μm rolling bins) relative to seizure wavefront. Photostimulation responses are Z scored to each target's distribution of baseline responses (one-way ANOVA: *****$p < 1e{-}11$). (Right) The average neuropil signal associated with all cells over distance (20 μm rolling bins) relative to the seizure wavefront. Neuropil measurements are taken at photostimulation trials (from 0 to 250 ms prior to stimulation) and Z scored to each cell's distribution of baseline measurements (one-way ANOVA: *****$p < 1e{-}5$).

(C) Average photostimulation response of non-targeted neurons during the interictal state (blue) and classified proximal (pink) and distal (gold) to the seizure wavefront ($N = 6$ mice; one-way ANOVA: *****$p < 1e{-}46$).

(D) Influence of photostimulation on the activity of non-targeted neurons proximal (left) and distal (right) to the seizure wavefront (one-way ANOVA: p(proximal) = 0.17, *p(distal) < 0.05).

Error bars and spans: mean ± 95% confidence interval (A and B) and mean ± SEM (C and D).

See also Figures S6 and S7.

to measure excitability at high-spatial resolution, we find that there is strong inhibition in close proximity to the seizure wavefront.

DISCUSSION

Combined optogenetics and calcium imaging (all-optical interrogation) enabled the measurement of single-neuron excitability in real time, *in vivo*, and simultaneously across many neurons. These studies showed that focal 4-AP injection leads to widescale hyperexcitability outside of the injection site, but this is not due to increased single-neuronal excitability. Yet, single-neuronal excitability is indeed dynamically modulated in the seizure penumbra relative to the seizure wavefront.

In the interictal period, widefield stimulation of neurons distal to 4-AP injection showed increased responses; however, 2P-targeted photostimulation of the same neurons found that single-neuronal excitability remained at baseline levels. Interestingly, though, there was an increase in the correlation of the dynamics of excitability between individual neurons. This suggests that neurons outside of the focus of 4-AP injection are hyperexcitable due to large, correlated pre-synaptic inputs rather than a change in post-synaptic excitability. Indeed, widefield stimulation was a strong trigger for ictal events, reflecting the suggestion that positive feedback loops underlie seizure onset.[28,29]

We also found that single-neuronal excitability was not affected leading up to seizure onset, but it was significantly elevated in the post-ictal period, a period that is marked by

generalized electrographic depression and decreased mental status in humans.[17,18] Our test finds that not only do neurons remain excitable following seizure termination but they can also respond at higher than baseline levels if directly stimulated. There is a concomitant post-ictal decrease in the widefield calcium signal below baseline levels, which is consistent with brain-wide suppression of neural activity. We propose that this widespread suppression creates a low-conductance state in neurons that leads to decreased shunting inhibition, thereby elevating neuronal excitability to single-neuron stimulation.

Interestingly, during seizure propagation, neurons distal to the seizure wavefront showed a generalized hyperexcitability. However, hypoexcitability was found in neurons just proximal to the seizure wavefront. This appears to be consistent with an activation of inhibitory neurons in concert with the propagating seizure wavefront. We propose that intense and synchronized firing of neurons in seizure creates a high post-synaptic conductance state (e.g., high GABAergic tonic conductance during seizures *in vitro*[30]) and that this leads to increased shunting of soma-targeted 2P stimulation of neurons in close proximity to the seizure wavefront that most directly receives these high-magnitude presynaptic inputs. Indeed, the span of relative hypoexcitability of ∼150 μm correlates well to the anatomically determined connection probability for inhibitory neurons in this region of the mouse cortex.[31,32]

Targeted photostimulation in the cortex consistently elicits more inhibition than excitation of downstream neurons.[20,21,25,26,33,34] However, during seizures, there is an intriguing contrast of hypoexcitability of directly photostimulated neurons with increased responses of downstream non-target neurons near the seizure wavefront. This suggests another possible effect of strong shunting inhibition, such that 2P-targeted excitation of neuronal somata is more sensitive to shunting than the propagated excitation, which reaches downstream neurons at dendrites. This leads to soma-targeted excitation being strongly reduced by shunting inhibition, but propagated downstream excitation at dendrites does not experience this powerful shunting inhibition. In the local proximity of a seizure wavefront, these non-target neurons also receive highly synchronized excitatory afferent drive from the wavefront itself, which likely further contributes to their increased responses.

Together, our results demonstrate that inhibitory neurons were activated in concert with—and not in advance of—the seizure wavefront. The cortex is known to be characterized by highly local anatomic and functional connectivity of inhibitory neurons.[20,22,26,31,32] This leads to a profile where highly active inhibitory neurons at the seizure wavefront impart strong local inhibition on excitatory neurons directly proximal to the seizure (Figure S7).

Limitations of the study and future directions

Although our imaging of Nkx2.1-labeled inhibitory neurons does not find that they are activated distal to the seizure wavefront, it may still be the case that inhibitory neurons are activated before excitatory neurons within the seizure wavefront. Calcium imaging does not afford temporal resolution to resolve this differentiation. Furthermore, the Nkx2.1-fate-mapping transgenic system does not label up to 40% of L2–4 PV and SST interneurons[15,16]; thus, it remains possible that a subset of interneurons are activated distal to the seizure wavefront. However, our direct measurement of excitability suggests that this is still not the case. Future studies can deploy our study's approach with emerging voltage imaging techniques to study cell-type-specific activity dynamics with high temporal resolution. We did not analyze the influence of the sex of the animals on the results of the study (animals of both sexes were used for this study).

Another key technical limitation of the study is the use of an acute model of focal seizures, which was necessary given the methodological requirement to study seizures in a predictable manner under a head-fixed microscopy setup. Thus, it will be important for future experiments to apply our methods in seizure propagation in models of chronic epilepsy.

STAR★METHODS

Detailed methods are provided in the online version of this paper and include the following:

- KEY RESOURCES TABLE
- RESOURCE AVAILABILITY
  - Lead contact
  - Materials availability
  - Data and code availability
- EXPERIMENTAL MODEL AND STUDY PARTICIPANT DETAILS
- METHOD DETAILS
  - Surgical preparation
  - Focal 4-AP acute seizures and local field potential recording
  - Awake head-fixed two-photon calcium imaging
  - Simultaneous widefield optogenetics and two-photon imaging
  - Simultaneous two-photon optogenetic stimulation and calcium imaging (all-optical interrogation)
- QUANTIFICATION AND STATISTICAL ANALYSIS
  - Analysis of two-photon calcium imaging and photostimulation experiments
  - Data processing
  - Analysis: Marking seizure onset and offset timing using LFP
  - Analysis: Interneuron calcium imaging
  - Analysis: Widefield photostimulation and two-photon imaging
  - Analysis: Ictal event-photostimulation correlation
  - Analysis: Measuring the seizure wavefront
  - Analysis: Photostimulation responses of targeted neurons
  - Analysis: Influence of photostimulation on non-targeted neurons

SUPPLEMENTAL INFORMATION

Supplemental information can be found online at https://doi.org/10.1016/j.celrep.2024.114189.

ACKNOWLEDGMENTS

We thank Richard Burman, Colin Akerman, Thijs van der Plas, Robert Lees, and James Rowland for useful discussions and technical support. This work was supported by funding from the Wellcome Trust (204651/Z/16/Z) to A.M.P., a Vanier - Canada Graduate Scholarship to P.T.S., Mitacs to P.T.S., and the University of Toronto MD/PhD Program to P.T.S.

AUTHOR CONTRIBUTIONS

P.T.S. performed animal surgeries. P.T.S., T.A.V., and A.M.P. designed the experiments. P.T.S. and A.M.P. performed the experiments. P.T.S. wrote the code for data analysis. P.T.S. and A.M.P. analyzed the data. P.T.S. and A.M.P. wrote the original manuscript. P.T.S., T.A.V., and A.M.P. reviewed

and edited the final manuscript. A.M.P. provided primary funding and resources. T.A.V. and A.M.P. supervised experiments and data analysis.

DECLARATION OF INTERESTS

The authors declare no competing interests.

Received: August 16, 2023
Revised: December 22, 2023
Accepted: April 17, 2024
Published: May 3, 2024

REFERENCES

1. Isaacson, J.S., and Scanziani, M. (2011). How Inhibition Shapes Cortical Activity. Neuron *72*, 231–243. https://doi.org/10.1016/j.neuron.2011.09.027.
2. Schevon, C.A., Weiss, S.A., McKhann, G., Goodman, R.R., Yuste, R., Emerson, R.G., and Trevelyan, A.J. (2012). Evidence of an inhibitory restraint of seizure activity in humans. Nat. Commun. *3*, 1060. https://doi.org/10.1038/ncomms2056.
3. Wenzel, M., Hamm, J.P., Peterka, D.S., and Yuste, R. (2017). Reliable and Elastic Propagation of Cortical Seizures In Vivo. Cell Rep. *19*, 2681–2693. https://doi.org/10.1016/j.celrep.2017.05.090.
4. Cammarota, M., Losi, G., Chiavegato, A., Zonta, M., and Carmignoto, G. (2013). Fast spiking interneuron control of seizure propagation in a cortical slice model of focal epilepsy: Fast-spiking interneurons in focal seizure propagation. J. Physiol. *591*, 807–822. https://doi.org/10.1113/jphysiol.2012.238154.
5. Liou, J.Y., Ma, H., Wenzel, M., Zhao, M., Baird-Daniel, E., Smith, E.H., Daniel, A., Emerson, R., Yuste, R., Schwartz, T.H., and Schevon, C.A. (2018). Role of inhibitory control in modulating focal seizure spread. Brain *141*, 2083–2097. https://doi.org/10.1093/brain/awy116.
6. Parrish, R.R., Codadu, N.K., Mackenzie-Gray Scott, C., and Trevelyan, A.J. (2019). Feedforward inhibition ahead of ictal wavefronts is provided by both parvalbumin- and somatostatin-expressing interneurons. J. Physiol. *597*, 2297–2314. https://doi.org/10.1113/JP277749.
7. Schevon, C.A., Tobochnik, S., Eissa, T., Merricks, E., Gill, B., Parrish, R.R., Bateman, L.M., McKhann, G.M., Emerson, R.G., and Trevelyan, A.J. (2019). Multiscale recordings reveal the dynamic spatial structure of human seizures. Neurobiol. Dis. *127*, 303–311. https://doi.org/10.1016/j.nbd.2019.03.015.
8. Schwartz, T.H., and Bonhoeffer, T. (2001). In vivo optical mapping of epileptic foci and surround inhibition in ferret cerebral cortex. Nat. Med. *7*, 1063–1067. https://doi.org/10.1038/nm0901-1063.
9. Sessolo, M., Marcon, I., Bovetti, S., Losi, G., Cammarota, M., Ratto, G.M., Fellin, T., and Carmignoto, G. (2015). Parvalbumin-Positive Inhibitory Interneurons Oppose Propagation But Favor Generation of Focal Epileptiform Activity. J. Neurosci. *35*, 9544–9557. https://doi.org/10.1523/JNEUROSCI.5117-14.2015.
10. Trevelyan, A.J., Sussillo, D., and Yuste, R. (2007). Feedforward Inhibition Contributes to the Control of Epileptiform Propagation Speed. J. Neurosci. *27*, 3383–3387. https://doi.org/10.1523/JNEUROSCI.0145-07.2007.
11. Trevelyan, A.J., and Schevon, C.A. (2013). How inhibition influences seizure propagation. Neuropharmacology *69*, 45–54. https://doi.org/10.1016/j.neuropharm.2012.06.015.
12. Trevelyan, A.J., Sussillo, D., Watson, B.O., and Yuste, R. (2006). Modular Propagation of Epileptiform Activity: Evidence for an Inhibitory Veto in Neocortex. J. Neurosci. *26*, 12447–12455. https://doi.org/10.1523/JNEUROSCI.2787-06.2006.
13. Wenzel, M., Hamm, J.P., Peterka, D.S., and Yuste, R. (2019). Acute Focal Seizures Start As Local Synchronizations of Neuronal Ensembles. J. Neurosci. *39*, 8562–8575. https://doi.org/10.1523/JNEUROSCI.3176-18.2019.
14. Shimoda, Y., Leite, M., Graham, R.T., Marvin, J.S., Hasseman, J., Kolb, I., Looger, L.L., Magloire, V., and Kullmann, D.M. (2023). Extracellular glutamate and GABA transients at the transition from interictal spiking to seizures. Preprint at bioRxiv. https://doi.org/10.1101/2020.11.13.381707.
15. Xu, Q., Tam, M., and Anderson, S.A. (2008). Fate mapping Nkx2.1-lineage cells in the mouse telencephalon. J. Comp. Neurol. *506*, 16–29. https://doi.org/10.1002/cne.21529.
16. Anastasiades, P.G., Marques-Smith, A., Lyngholm, D., Lickiss, T., Raffiq, S., Kätzel, D., Miesenböck, G., and Butt, S.J.B. (2016). GABAergic interneurons form transient layer-specific circuits in early postnatal neocortex. Nat. Commun. *7*, 10584. https://doi.org/10.1038/ncomms10584.
17. Bauer, P.R., Thijs, R.D., Lamberts, R.J., Velis, D.N., Visser, G.H., Tolner, E.A., Sander, J.W., Lopes da Silva, F.H., and Kalitzin, S.N. (2017). Dynamics of convulsive seizure termination and postictal generalized EEG suppression. Brain *140*, 655–668. https://doi.org/10.1093/brain/aww322.
18. So, N.K., and Blume, W.T. (2010). The postictal EEG. Epilepsy Behav. *19*, 121–126. https://doi.org/10.1016/j.yebeh.2010.06.033.
19. Packer, A.M., Russell, L.E., Dalgleish, H.W.P., and Häusser, M. (2015). Simultaneous all-optical manipulation and recording of neural circuit activity with cellular resolution in vivo. Nat. Methods *12*, 140–146. https://doi.org/10.1038/nmeth.3217.
20. Chettih, S.N., and Harvey, C.D. (2019). Single-neuron perturbations reveal feature-specific competition in V1. Nature *567*, 334–340. https://doi.org/10.1038/s41586-019-0997-6.
21. Dalgleish, H.W., Russell, L.E., Packer, A.M., Roth, A., Gauld, O.M., Greenstreet, F., Thompson, E.J., and Häusser, M. (2020). How many neurons are sufficient for perception of cortical activity? Elife *9*, e58889. https://doi.org/10.7554/eLife.58889.
22. Fino, E., Packer, A.M., and Yuste, R. (2013). The logic of inhibitory connectivity in the neocortex. Neuroscientist *19*, 228–237. https://doi.org/10.1177/1073858412456743.
23. Holmgren, C., Harkany, T., Svennenfors, B., and Zilberter, Y. (2003). Pyramidal cell communication within local networks in layer 2/3 of rat neocortex. J. Physiol. *551*, 139–153. https://doi.org/10.1113/jphysiol.2003.044784.
24. Kapfer, C., Glickfeld, L.L., Atallah, B.V., and Scanziani, M. (2007). Supralinear increase of recurrent inhibition during sparse activity in the somatosensory cortex. Nat. Neurosci. *10*, 743–753. https://doi.org/10.1038/nn1909.
25. Russell, L.E., Fişek, M., Yang, Z., Tan, L.P., Packer, A.M., Dalgleish, H.W.P., Chettih, S.N., Harvey, C.D., and Häusser, M. (2024). The influence of cortical activity on perception. depends on behavioral state and sensory context. Nat. Commun. *15*, 2456. https://doi.org/10.1038/s41467-024-46484-5.
26. Oldenburg, I.A., Hendricks, W.D., Handy, G., Shamardani, K., Bounds, H.A., Doiron, B., and Adesnik, H. (2024). The logic of recurrent circuits in the primary visual cortex. Nat. Neurosci. *27*, 137–147. https://doi.org/10.1038/s41593-023-01510-5.
27. Mardinly, A.R., Oldenburg, I.A., Pégard, N.C., Sridharan, S., Lyall, E.H., Chesnov, K., Brohawn, S.G., Waller, L., and Adesnik, H. (2018). Precise multimodal optical control of neural ensemble activity. Nat. Neurosci. *21*, 881–893. https://doi.org/10.1038/s41593-018-0139-8.
28. Trevelyan, A.J., Graham, R.T., Parrish, R.R., and Codadu, N.K. (2023). Synergistic Positive Feedback Mechanisms Underlying Seizure Initiation. Epilepsy Curr. *23*, 38–43. https://doi.org/10.1177/15357597221127163.
29. Graham, R.T., Parrish, R.R., Alberio, L., Johnson, E.L., Owens, L., and Trevelyan, A.J. (2023). Optogenetic stimulation reveals a latent tipping point in cortical networks during ictogenesis. Brain *146*, 2814–2827. https://doi.org/10.1093/brain/awac487.
30. Magloire, V., Savtchenko, L.P., Jensen, T.P., Sylantyev, S., Kopach, O., Cole, N., Tyurikova, O., Kullmann, D.M., Walker, M.C., Marvin, J.S.,

CellPress
OPEN ACCESS

et al. (2023). Volume-transmitted GABA waves pace epileptiform rhythms in the hippocampal network. Curr. Biol. *33*, 1249–1264.e7. https://doi.org/10.1016/j.cub.2023.02.051.

31. Packer, A.M., and Yuste, R. (2011). Dense, Unspecific Connectivity of Neocortical Parvalbumin-Positive Interneurons: A Canonical Microcircuit for Inhibition? J. Neurosci. *31*, 13260–13271. https://doi.org/10.1523/JNEUROSCI.3131-11.2011.
32. Campagnola, L., Seeman, S.C., Chartrand, T., Kim, L., Hoggarth, A., Gamlin, C., Ito, S., Trinh, J., Davoudian, P., Radaelli, C., et al. (2022). Local connectivity and synaptic dynamics in mouse and human neocortex. Science *375*, eabj5861. https://doi.org/10.1126/science.abj5861.
33. Gauld, O.M., Packer, A.M., Russell, L.E., Dalgleish, H.W.P., Iuga, M., Sacadura, F., Roth, A., Clark, B.A., and Häusser, M. (2024). A latent pool of neurons silenced by sensory-evoked inhibition can be recruited to enhance perception.. Preprint at bioRxiv. https://doi.org/10.1101/2024.02.12.579847.
34. Rowland, J.M., Van Der Plas, T.L., Loidolt, M., Lees, R.M., Keeling, J., Dehning, J., Akam, T., Priesemann, V., and Packer, A.M. (2023). Propagation of activity through the cortical hierarchy and perception are determined by neural variability. Nat. Neurosci. *26*, 1584–1594. https://doi.org/10.1038/s41593-023-01413-5.
35. Dana, H., Sun, Y., Mohar, B., Hulse, B.K., Kerlin, A.M., Hasseman, J.P., Tsegaye, G., Tsang, A., Wong, A., Patel, R., et al. (2019). High-performance calcium sensors for imaging activity in neuronal populations and microcompartments. Nat. Methods *16*, 649–657. https://doi.org/10.1038/s41592-019-0435-6.
36. Chen, T.-W., Wardill, T.J., Sun, Y., Pulver, S.R., Renninger, S.L., Baohan, A., Schreiter, E.R., Kerr, R.A., Orger, M.B., Jayaraman, V., et al. (2013). Ultrasensitive fluorescent proteins for imaging neuronal activity. Nature *499*, 295–300. https://doi.org/10.1038/nature12354.
37. Watson, B.O., Yuste, R., and Packer, A.M. (2016). PackIO and EphysViewer: software tools for acquisition and analysis of neuroscience data. Preprint at bioRxiv. https://doi.org/10.1101/054080.
38. Russell, L.E., Dalgleish, H.W.P., Nutbrown, R., Gauld, O.M., Herrmann, D., Fişek, M., Packer, A.M., and Häusser, M. (2022). All-optical interrogation of neural circuits in behaving mice. Nat. Protoc. *17*, 1579–1620. https://doi.org/10.1038/s41596-022-00691-w.
39. Pachitariu, M., Stringer, C., Dipoppa, M., Schröder, S., Rossi, L.F., Dalgleish, H., Carandini, M., and Harris, K.D. (2016). Suite2p: beyond 10,000 neurons with standard two-photon microscopy. Preprint at bioRxiv. https://doi.org/10.1101/061507.
40. Stringer, C., Wang, T., Michaelos, M., and Pachitariu, M. (2021). Cellpose: a generalist algorithm for cellular segmentation. Nat. Methods *18*, 100–106. https://doi.org/10.1038/s41592-020-01018-x.
41. Schneider, C.A., Rasband, W.S., and Eliceiri, K.W. (2012). NIH Image to ImageJ: 25 years of image analysis. Nat. Methods *9*, 671–675. https://doi.org/10.1038/nmeth.2089.
42. Gerchberg, R.W., and Saxton, W.O. (1972). A Practical Algorithm for the Determination of Phase from Image and Diffiraction Plane Pictures. Optik *35*, 237–246.
43. Guo, L., Rivero, D., Dorado, J., Rabuñal, J.R., and Pazos, A. (2010). Automatic epileptic seizure detection in EEGs based on line length feature and artificial neural networks. J. Neurosci. Methods *191*, 101–109. https://doi.org/10.1016/j.jneumeth.2010.05.020.
44. Rossi, L.F., Wykes, R.C., Kullmann, D.M., and Carandini, M. (2017). Focal cortical seizures start as standing waves and propagate respecting homotopic connectivity. Nat. Commun. *8*, 217. https://doi.org/10.1038/s41467-017-00159-6.

STAR★METHODS

KEY RESOURCES TABLE

REAGENT or RESOURCE	SOURCE	IDENTIFIER
**Bacterial and virus strains**		
syn-GCaMP7f	Dana et al.[35]	AAV2/1-syn-jGCaMP7f-WPRE; RRID:Addgene_104488
C1V1-Kv2.1	Chettih and Harvey[20]	AAV2/9-CaMKIIa-C1V1-t/t-kv2.1-mScarlet; RRID: Addgene_124650
syn-GCaMP6s	Chen et al.[36]	AAV2/1-syn-GCaMP6s-WPRE-SV40; RRID: Addgene_100843
**Chemicals, peptides, and recombinant proteins**		
4-aminopyridine	Torcis	#0940
Silicone	Kwik-cast	N/A
Superbond C&B Dental Cement	Sun Medical	K058E
Buprenorphine (Vetergesic)	Ceva Animal Health Ltd.	N/A
Meloxicam (Metacam)	Boehringer Ingelheim	N/A
Chlorhexidine gluconate and Isopropyl alcohol (CareFusion Chloraprep)	Williams Medical Supplies Ltd	D56177
Cyanoacrylate glue (3M Vetbond)	Vet Direct	VB3M
**Experimental models: Organisms/strains**		
Wildtype	Charles-River Laboratories	C57BL/6
Camklla-tTa:GCaMP6s	Charles-River Laboratories	CamKIIa-tTa(AI94) x DBA-Tg(tetO-GCaMP6s)2Niell/J
Nkx2.1::tdTomato	Charles-River Laboratories	Nkx2.1-Cre x tdTomato (AI9)
**Software and algorithms**		
Imaging+	https://github.com/Packer-Lab/imagingplus	https://doi.org/10.5281/zenodo.10825258
All original code	This study; https://github.com/Packer-Lab/AllOpticalSeizure	https://doi.org/10.5281/zenodo.10901365
PackIO	Watson et al.[37]; http://apacker83.github.io/PackIO/	N/A
NAPARM	Russell et al.[38]; https://github.com/llerussell/Naparm	N/A
Suite2p	Pachitariu et al.[39]; https://www.suite2p.org	v0.11.1
Cellpose	Stringer et al.[40]; https://www.cellpose.org	v2.1.1
ImageJ	Schneider et al.[41]	v2.0.0
scipy	Anaconda	v1.8.1
Blink SDK	Medowlark Optics	v1.0.527.1
PrairieView	Bruker Corp.	v5.5.1rev203
STAMovieMaker	https://github.com/llerussell/STAMovieMaker	N/A
Python	Anaconda	3.8, 3.9
pycircstat	https://github.com/circstat/pycircstat	N/A
**Other**		
Stereotaxic frame	Stoelting	51733D
Hydraulic micromanipulator	Narishige	MMO-220A
Micromanipulator	Luigs & Neumann	LN Mini Compact
Amplifier	Axon instruments	700B

(*Continued on next page*)

CellPress
OPEN ACCESS

Continued

REAGENT or RESOURCE	SOURCE	IDENTIFIER
Resonant scanning microscope	Bruker Corp.	2pPlus
Tunable laser	Coherent	Vision-S
Fiber laser (1030nm)	Coherent	Monaco
Pockels cell	Conoptics	N/A
Blue light LED	Thorlabs	M470L3
LED driver	Thorlabs	LEDD1B
512x512 Spatial Light Modulator (SLM)	Boulder Nonlinear Systems	N/A
Dental drill	NSK UK Ltd	N/A
USB data acquisition I/O card	National Instruments	PCI-6052E

RESOURCE AVAILABILITY

Lead contact

Further information and requests for resources and reagents should be directed to and will be fulfilled by the lead contact, Adam M. Packer (adampacker@gmail.com).

Materials availability

There are no relevant materials produced in this manuscript.

Data and code availability

- Pre-processed imaging data reported in this paper will be shared by the lead contact upon request.
- All original code has been deposited at https://github.com/Packer-Lab/AllOpticalSeizure and at https://github.com/Packer-Lab/imagingplus, and is publicly available as of the date of publication. Any additional information required to reanalyze the data reported in this paper is available from the lead contact upon request.

EXPERIMENTAL MODEL AND STUDY PARTICIPANT DETAILS

Animal experiments were carried out in accordance with the guidelines and regulations of the UK Home Office (Animals in Scientific Procedures Act of 1986). Animal Welfare Ethical Review Body of the University of Oxford approved animal experiments. Adult mice of both sexes (2–6 months of age) on a C57BL/6 background were used for experiments. Four wildtype and seven GCaMP6s transgenic mice (CamKIIa-tTa(AI94) x B6; DBA-Tg(tetO-GCaMP6s)2Niell/J) were used for imaging of seizures and all-optical interrogation experiments. Additionally, four Nkx2.1-Cre x tdTomato (AI9) were used for inhibitory neuron imaging experiments. All mice were housed in groups of up to 4 per cage at room temperature (20–22°C) on a standard light/dark cycle and humidity of ~40%, and provided with a standard mouse chow diet and received daily husbandry. Mice who underwent surgical preparation for imaging experiments were housed together.

METHOD DETAILS

Surgical preparation

To prepare mice for imaging experiments, mice underwent a single surgery consisting of headplate implantation, cranial window implantation and viral injection. Mice were anesthetized with isoflurane (5% for induction and 1%–2% for maintenance), and subsequently mounted into a heated stereotaxic frame. A perioperative injection of 0.1 mg/kg buprenorphine (Vetergesic), 5 mg/kg meloxicam (Metacam) was administered. The scalp was sterilized using chlorhexidine gluconate and isopropyl alcohol (ChloraPrep) and subsequently a midline incision was made to expose the skull. After removing the scalp bilaterally, the location of the cranial window implant was stereotaxically marked (right somatosensory cortex: −1.9mm AP, +3.8 mm ML from bregma). Then, a custom-machined aluminum headplate with a 7mm imaging well was secured on the skull using dental cement (C + B Metabond). A 3mm circular craniotomy was drilled centered on the previously marked location and the skull removed after soaking with saline. The dura overlying the exposed cortex was also carefully removed under regular saline flushing.

After achieving control over any actively bleeding blood vessels, 800nL injection of a diluted virus mixture was performed. The viral injection mixture for opsin expression in transgenic GCaMP6s and wildtype mice consisted of C1V1-Kv2.1 (AAV2/9-CaMKIIa-C1V1-t/t-kv2.1-mScarlet; approximately 2e12 GC/ml diluted 1:5). The viral injection for wildtype mice also additionally contained GCaMP6s (AAV2/1-*syn*-GCaMP6s-WPRE-SV40; approximately 2.5e13 GC/ml diluted 1:10 in sterile phosphate-buffered saline). Nkx2.1-tdTomato mice received GCaMP7f (AAV2/1-*syn*-jGCaMP7f-WPRE; diluted 1:20 in sterile phosphate-buffered saline). All viral

injections were performed at 300 μm below the pial surface through a pulled glass injection needle and a hydraulic nanoinjector (Narishige) at a rate of 100 nL/min; the needle was left in following each injection for 10 min to allow diffusion of virus.

Finally, a cranial window, consisting of two no. 1 thickness coverglass (3 mm and 4 mm diameter optically adhered to one another), was implanted over the craniotomy and fully sealed using cyanoacrylate (VetBond) and Metabond dental cement. The cranial window was pre-drilled with an 800 μm hole in the corner to allow access for 4-AP injection and LFP recording pipettes. This hole was offset to the edge of the cranial window to ensure the 4-AP injection and LFP recording locations were positioned away from the center of the window where viral injection was delivered. The hole in the cranial window was filled using a silicone Kwik-cast plug before implanting over the craniotomy. Mice received buprenorphine at the time of surgery and in food gels for 2 days post-surgery. All animals were given 3 weeks to recover and reach optimal viral expression before experimentation.

Focal 4-AP acute seizures and local field potential recording

Focal injection of 4-aminopyridine (4-AP) was used to produce repeated acute seizures of focal-onset in mice and nearby local field potential (LFP) signal was used to electrophysiologically record seizures for the duration of the experiment.

The Kwik-cast plug sealing the pre-drilled access hole of the cranial window was removed after setting up mice in head-fixed setup. 4-AP (5mM) was loaded into a glass pulled pipette mounted on a micromanipulator which was then lowered into the cortex through the access hole in the cranial window under 5x widefield imaging. 4-AP was injected as a bolus of 200nL using a hydraulic nanoinjector (Narishige) at a rate of 400 nL/min. Up to 2 more bolus injections would be made if there was no seizure for 15 min. A second glass pulled pipette, backfilled with 45 μm filtered cortex buffer solution (contain in mM: 125 NaCl, 5 KCl, 10 Glucose, 10 HEPES, 2 CaCl2, 2 MgSO4), was inserted in parallel to the 4-AP injection pipette using a second micromanipulator to approximately the same depth and location. LFP signal was collected from this pipette through a headstage, Axon 700B amplifier (200Hz Bessel filter), a 50/60Hz noise-cancellation humbug, and digitised at 20kHz using PackIO (http://apacker83.github.io/PackIO/, a software for electrophysiology data acquisition, digitization and synchronized triggering of instruments). After successful insertion of both pipettes, they were not moved for the entire duration of the experiment and the Nikon 16x objective was lowered into position under water immersion to begin 2P imaging experiments.

Awake head-fixed two-photon calcium imaging

2P imaging was performed using a resonant scanning microscope (2pPlus, Bruker Corporation), a tunable femtosecond-pulsed, dispersion-compensated laser beam (Vison-S, Coherent) and a 16x/0.8-NA water-immersion objective lens (Nikon). Total power was modulated with a Pockels cell (Conoptics) and maintained at or below 50mW on sample for all experiments. The primary dichroic (Chroma ZT473-491/NIRpc) split 2P excitation and emission light. Emission light was passed through a green/red splitting dichroic (Chroma T560lpxr), then GCaMP6s and GCaMP7f imaging was performed using a 920nm beam and a green-light collection filter set (Chroma ET525/50m-2p), and mCherry or tdTomato imaging was performed using a 765nm beam and a red-light collection filter set (Chroma ET595/50m-2p). All animals were headfixed under the experimental imaging setup to confirm expression of virally-transfected calcium indicator and/or opsin at least once prior to experimentation. The cortical location for 2P imaging and all-optical experimentation under the cranial window was selected at least 1 mm away from insertion of 4-AP injection and LFP recording pipettes. 2P imaging was performed in Layer 2/3 at a depth of 150–300 μm below the pia depending on optimal GCaMP6s and mCherry (for C1V1-Kv2.1) expression. For 2P calcium imaging of interneurons in Nkx2.1-Cre-tdTomato mice, an FOV was selected at the start of the experiment at least 1 mm away from the focal 4-AP injection and LFP recording site and with optimal co-expression of GCaMP7f virus with tdTomato labeled interneurons. Live 2P imaging was performed at either 15Hz (1024 x 1024 pixels; $N = 4$ experiments) or 30Hz (512 x 512 pixels; $N = 2$ experiments) and at between 0.8x to 1.5x zoom. Imaging was controlled through PrairieView (Bruker Corporation).

All experiments were terminated either when the experimental animal entered status epilepticus or the total experiment duration reached 3hrs, whichever occurred first.

Simultaneous widefield optogenetics and two-photon imaging

Widefield (1P) optogenetic stimulation was performed during resonant-scanning 2P calcium imaging using the epi-illumination module of the Bruker 2pPlus. In particular, widefield blue light passed through the 2P excitation light path was delivered to the FOV using a blue light LED (Thorlabs M470L3) driven by a LED driver (Thorlabs LEDD1B) in trigger mode. This blue light for widefield optogenetic stimulation was passed through the 2P imaging dichroic (Chroma ZT473-491/NIRpc, which also contained a notch for blue light reflectance in addition to near-infrared light reflectance) to reach the FOV. Blue light photostimulation was delivered as 10 × 10ms pulses @ 25Hz with 3.5mW power on sample. To avoid damage to the 2P imaging photomultiplier tubes (PMTs) from widefield blue light exposure, high-speed shutters on all PMTs were triggered during widefield photostimulation. Triggers for blue light stimulation and the high-speed PMT shutters were provided using PackIO.

Simultaneous two-photon optogenetic stimulation and calcium imaging (all-optical interrogation)

2P all-optical experiments were performed by simultaneous 2P stimulation of selected neurons using a spatial light modulator (SLM) and calcium imaging (as described above) in the same FOV using two independent beam paths setup in the Bruker 2pPlus. 2P optogenetic stimulation was performed using a pulsed fixed-wavelength fiber laser 1035nm (Monaco, Coherent) at a repetition rate of

2 MHz. Multiple individual neurons were simultaneously targeted for stimulation by splitting the laser beam into beamlets using a reflective spatial light modulator (SLM) (7.68 × 7.68 mm active area, 512 x 512 pixels, Boulder Nonlinear Systems). The active area of the SLM was overfilled and the polarisation optimised for maximal first order diffraction efficiency using a half-wave plate. The zero order diffraction beam was blocked using a well bored into an optical flat using a dental drill (NSK UK Ltd).

SLM phase masks were loaded using the Blink SDK (Medowlark Optics). Phase masks were computed by applying the Gerchberg-Saxton algorithm to the *xy* coordinates of the targets.[42] A weighted form of this algorithm was used to ensure uniform power distribution (6mW/target) across all targets to account for reduction in the first order diffraction efficiency of the SLM with increasing distance from the zero order location. An image of the SLM was relayed onto a pair of galvanometer mirrors (independent of the imaging beam path) integrated in the 2P imaging system. The galvanometer mirrors were programmed to generate spirals of 10 μm diameter (10 × 20ms pulses @ 40Hz) by moving all beamlets simultaneously.

The affine transformation required to map coordinates from SLM space to imaging space was computed through a custom-modified version of *NAPARM*[38] (github.com/llerussell/Naparm) and calibrated at the start of each experiment by burning arbitrary patterns into a fluorescent plastic slide. Phase masks and galvanometer voltages required to perform photostimulation were generated using *NAPARM*.[38] Voltages were applied to the photostimulation-path galvanometers using PrairieView (Bruker Corporation). A USB data acquisition card (National Instruments) running PackIO, was used as a master synchroniser to record individual signals representing the LFP electrophysiological signal, the frame clock of 2P imaging and various photostimulation signals (e.g., galvanometer voltages, SLM phase mask trigger and voltages).

In some experiments, a single widefield blue light stimulation trial driven using PackIO was used to test the optogenetic photostimulation-induced calcium response while exploring the FOV and selecting the optimal region of fluorescence responsive neurons. A photostimulation-cell mapping protocol was run in all-optical experiments and online analysis of 2P photostimulation was carried out using STAMovieMaker (https://github.com/llerussell/STAMovieMaker) to test for responsive targets. These images were used to manually select the coordinates of neurons within the FOV for targeting of 2P optogenetic stimulation.

QUANTIFICATION AND STATISTICAL ANALYSIS

Analysis of two-photon calcium imaging and photostimulation experiments

Imaging data analysis was carried out in Python 3.8 and Python 3.9. Overall data analysis and procedures extended upon the framework provided in *Imaging+* (https://imagingplus.readthedocs.io/en/latest/), a newly developed Python package for the analysis of multimodal, time-synced, live-imaging neuroscience experiments. We excluded experiments where onset/offset timings of seizure events could not be determined from the LFP recording. We also excluded one experiment where seizure propagation did not propagate throughout a significant portion of the imaging FOV.

Data processing

All calcium imaging movies were first processed through the *Suite2p* pipeline[39] for movement correction and cell segmentation, followed by manual curation of Suite2p generated cell masks. Synchronised timeseries data signals collected as individual channels in PackIO (e.g., LFP signal, photostimulation hardware triggers) were processed using the *paq* processing submodule of *Imaging+*. The photostimulation trigger timestamp and the total duration of the photostimulation protocol were used to define each photostimulation trial's onset and offset. The photostimulation onset/offset timestamps were cross-referenced to the imaging frame clock signal to define photostimulation onset and offset in the imaging data. We excluded imaging frames between onset and offset of each photostimulation trial and all imaging frames from imaging trials after 4-AP injection from cell segmentation as photostimulation laser induced imaging artifacts and aberrant neuronal activity, respectively, confounded Suite2p cell segmentation performance. Fluorescent traces were recovered from photostimulated targeted neurons using a 10 μm diameter circular mask centered on each SLM target coordinate within the FOV. dFF normalization was performed on all traces, in which the whole trace was normalized to the mean of the trace (calculated with photostimulation artifact and ictal-event frames removed).

Analysis: Marking seizure onset and offset timing using LFP

The raw, digitized LFP signal was used for manually marking seizure onset/offset timings. No signal processing was performed on the LFP signal during data analysis. There are generally two electrographic patterns of seizure onset: hypersynchronous and low-voltage fast. Both result in an increase in the LFP power, allowing for automatic seizure onset and offset detection methods. Wenzel et al.,[3,13] use a threshold of LFP >5 SD from the interictal mean LFP power. In addition, there are also more advanced statistical models for seizure detection, but the main goal of these models remains to detect well-recognized features of the onset of seizures.[43] The primary benefit of such automatic methods is to allow for a consistent timing of seizure onset and offset with high temporal resolution. Although we initially tested the threshold method for automatic detection of seizure onset and offset, we found it was not sufficiently sensitive to detect all seizure events if the interictal LFP demonstrated a high variability. Since each animal replicate contained only a limited number of seizure events that could be used for imaging analysis, and because the primary goal of this phase of the analysis was to identify the photostimulation trials that occurred during seizure, and the inter-photostimulation trial period was >5 s, a high temporal resolution timing of seizure onset and offset was not required. Thus, we manually studied all experiments and classified seizure onset as the last sharp deflection in the LFP signal before a sustained barrage of ictal discharges. Seizure offset was defined

at the end of final large deflection in the LFP signal following a period of ictal discharges. All photostimulation trials outside of seizure onset/offset timings were classified as 'interictal', and those within as 'ictal'. In some cases, the photostimulation experiment was initiated during an ongoing seizure, and therefore all photostimulation trials until the offset of the initial seizure were classified as 'ictal'. No seizure onset was defined for these seizures.

Analysis: Interneuron calcium imaging

Inhibitory and excitatory neurons were defined as tdTomato+ve and tdTomato-ve cells, respectively, in virally-transfected GCaMP7f imaging in Nkx2.1-Cre-tdTomato transgenic mice. Baseline imaging trials (spontaneous activity, before 4-AP injection) were processed through Suite2p to generate motion-corrected imaging data and segmented ROIs for analysis. The tdTomato image of the FOV was co-registered (using *pystackreg*) to the motion-corrected Suite2p output image with a transform matrix that was calculated by registering a simultaneously collected GCaMP image to the registered image output obtained from Suite2p.

The Suite2p segmented ROIs were used to define excitatory neurons in the FOV and Cellpose[40] was used to automatically define cellular ROIs for inhibitory neuron from the co-registered tdTomato image. Suite2p ROIs with greater than 5% overlap in pixels with any inhibitory neuron ROIs were excluded from further analysis. Neuropil subtraction was performed on each respective Suite2p ROI's signal. To analyze the relationship for seizure recruitment between an inhibitory neuron and its local surrounding region, an annulus with an inner radius of 20 μm and outer radius of 100 μm was created from the center of the inhibitory neuron. This area was used to collect the average local signal from motion-corrected Suite2p imaging frames. Seizure recruitment for both inhibitory neuron and the local surrounding region was defined as time post-seizure onset (defined using the simultaneously collected LFP signal) to reach 65% of the maximum fluorescence signal during the seizure, as per previous calcium imaging studies of seizure propagation.[44]

Analysis: Widefield photostimulation and two-photon imaging

Widefield photostimulation timings were retrieved from the high-speed shutter loopback signal collected as a temporally synchronised PackIO channel. Although the high-speed shutters were deployed slightly before and released slightly after the LED photostimulation, there is no useful imaging data during the activation of the high-speed shutter. The coarse fluorescence calcium imaging signal was collected as the mean of the FOV from raw imaging movies. Photostimulation trial response magnitudes were quantified as the mean post-stimulation signal (collected from 0ms to +500ms post-high-speed shutter release) minus the mean pre-stimulation signal (collected from −500ms to 0ms pre-high-speed shutter deployment). The decay constant of the evoked photostimulation response was calculated as the time post-phototstimulation when the mean FOV fluorescence signal decreased to below 63% of the maximum post-stimulation value. Photostimulation trials in which post-stimulation fluorescence trace did not return to below this threshold (e.g., when photostimulation evoked ictal events) were excluded from this quantification.

Analysis: Ictal event-photostimulation correlation

The correlation of photostimulation and ictal onset in widefield photostimulation and holographic photostimulation experiments was calculated using the *pycircstat* package (https://github.com/circstat/pycircstat). The inter-photostimulation time period was divided into phase space of 1 s bins (0sec bin: −500ms to +500ms relative to photostimulation). The number of seizure event onset in each photostimulation phase bin was collected and the V-test for circular non-uniformity was performed with a mean direction of 0rads.

Analysis: Measuring the seizure wavefront

The location of the seizure wavefront was manually assessed for all 'ictal' photostimulation trials based on a 100-frame averaged image centered on each individual photostimulation trial. The frame averaged image gave a low-noise image for better visualization of the location of the seizure wavefront at the time of the photostimulation trial. This image was then studied using ImageJ to determine the seizure wavefront, which was visually assessed where there was a drop in the calcium fluorescence signal between the seizure core and the seizure penumbra. Then, we placed two coordinates (at least 100 μm apart) across the image to denote the leading edge of the seizure wavefront. A boundary of the seizure wavefront was linearly approximated using a line projection across the image constrained by these two coordinates. A line boundary was calculated for each photostimulation trial and used to calculate the shortest linear distance between all neurons to the seizure wavefront at each photostimulation trial.

Within each individual seizure event, the time of seizure invasion was individually marked for each photostimulation targeted neuron. This was marked at the initiation of the greatest upward deflection in the raw fluorescence signal that led to the maximum fluorescence signal during that event for that neuron. Then, a delay to seizure invasion was calculated at each photostimulation trial using this marked timing for each neuron.

Analysis: Photostimulation responses of targeted neurons

Responses of photostimulation-target neurons on photostimulation trials were collected from circular areas defined within the FOV using the spatial photostimulation target area outputs from *NAPARM*. These areas were applied to the motion corrected movie output from Suite2p processing to collect the average raw calcium fluorescence signal from pixels within each target. Photostimulation response magnitudes for each target were calculated from dFF normalized values by subtracting the average

pre-photostimulation signal (from −500 ms to photostimulation onset) from the mean post-photostimulation signal (from photostimulation offset to +500 ms). This procedure was applied equally across all baseline and post-4AP photostimulation trials.

The variability of photostimulation responses for each neuron was calculated as the coefficient of variation of response magnitudes from all photostimulation trials belonging to the appropriate state. *Z-scoring* was used to generate normalized photostimulation responses for single neurons. The underlying data (i.e., raw photostimulation-response magnitudes of single neurons) from each experiment satisfied the condition of normality necessary for z-scoring (statistical test of normality (*scipy*): p(for each exp.) < 1e-12). Each photostimulation trial of each neuron was *z-scored* to that neuron's total distribution of all photostimulation responses from the baseline state. This converted raw photostimulation responses to normalized z-scores for any given neuron and photostimulation trial (across any stage of the experiment).

Analysis: Influence of photostimulation on non-targeted neurons

The influence metric, originally designed in Chettih and Harvey (2019),[20] is a method for measuring the change in activity of each non-targeted neuron following photostimulation of a separate photostimulation-targeted neuron. A positive influence value between two neurons suggests that the photostimulation-target neuron has a net-excitatory impact on the non-target neuron, whereas a negative influence value suggests that the photostimulation-target has a net-inhibitory impact on the non-target neuron.

The influence metric is calculated as the difference in the response on individual photostimulation trials (photostimulation + visual stimulation) from the average of all control trials (visual stimulation only), normalized by the standard deviation of this difference over all trials. The resulting value quantifies, for a given single photostimulation trial, the relationship between the activity of a photostimulated target neuron on another non-target (non-photostimulated) neuron.

In our dataset, non-targeted neurons were selected from the overall curated set of ROIs generated by Suite2p. A circular exclusion zone with radius of 20 μm was defined around the center of each targeted neuron's coordinate in the FOV to exclude any potential Suite2p ROIs that may have received off-target activation during photostimulation. Photostimulation responses for non-target neurons were calculated similarly to the procedure used for target neurons. To measure the relationship between photostimulation response magnitude of targets and the photostimulation response magnitude of non-targets, the total photostimulation response magnitude of all non-targets was plotted against the total photostimulation response magnitude of targeted neurons for each trial. These values were *z-scored* within each experimental replicate to allow comparisons across all replicates. A similar procedure was also performed on "artificial stimulation" trials. These were artificially created stimulation trials defined during analysis as an alternative to sham (no-photostimulation) trials. These trials were interleaved between photostimulation trials. The responses to artificially defined stimulation trials were calculated and analyzed similarly to photostimulation trials to measure the relationship between normalized total response of targets vs. total response of non-targets during artificial stimulation trials.

To measure the influence of photostimulation of targeted neurons on non-targeted neurons, we needed to use a modified version of the influence metric given the differences in experimental setup between the present data and the data in Chettih and Harvey (2019)[20]: In order to measure the influence relative to a given photostimulation trial, it is necessary to first quantify a predicted level of activity for each non-targeted neuron. Then, the measured photostimulation trial response is normalized against this predicted response level to generate a photostimulation-driven influence metric for a given non-target neuron.

In Chettih and Harvey (2019),[20] this predicted level of non-targeted activity is inferred from non-photostimulation trials. However, such trials were not available in our experimental dataset. Instead, based on our finding (Figure S5) that the photostimulation responses of non-targeted neurons are correlated to each other within each photostimulation trial, we used the mean photostimulation response of all non-targeted neurons as the predicted response magnitude for all non-target neurons in a trial-wise manner.

Cell Reports, Volume *43*

Supplemental information

Highly local activation of inhibition at the seizure wavefront *in vivo*

Prajay T. Shah, Taufik A. Valiante, and Adam M. Packer

Supplemental Information

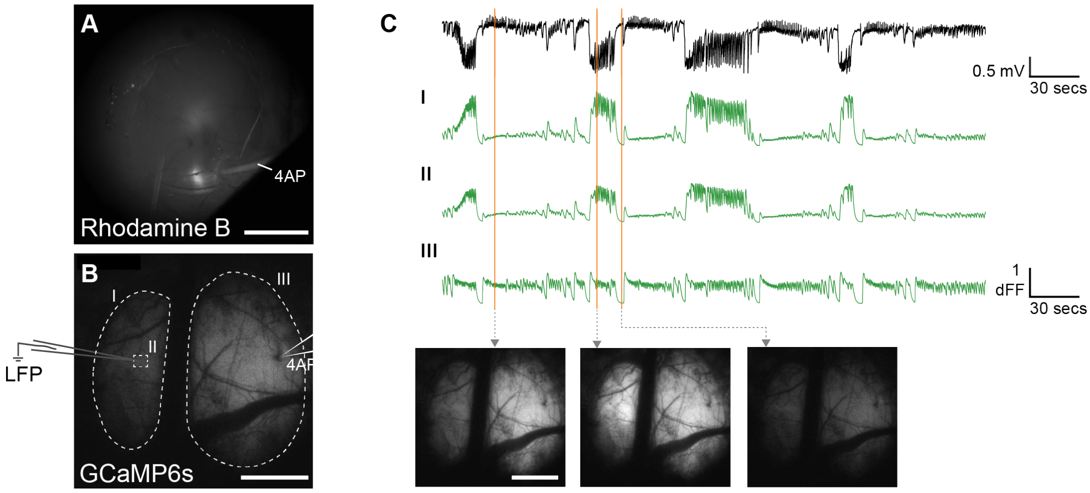

Figure S1. Cross-hemispheric, widefield calcium imaging of focal onset seizure. Related to Figure 1.

A. Focal 4-AP injection (mixed with Rhodamine-B) near the edge of the implanted cranial window. Note the fluorescence signal at the centre of the cranial window in this imaging channel is from viral-injection-transduced expression of C1V1. Scale bar = 1 mm

B. Widefield, cross-hemispheric calcium imaging under a craniotomy over the frontal cortex. LFP signal was collected contralateral to the focal 4-AP injection. ROIs were selected for fluorescence signal collection relative to the 4-AP injection and LFP recording electrode (I, II and III). Scale bar = 1 mm

C. Average fluorescence signal (green traces) from various ROIs marked in panel B, synchronised to the simultaneously collected LFP trace (black trace). Below is shown single-frame widefield calcium images during interictal, ictal and following seizure termination. Orange lines represent time points of imaging frames shown below. Scale bar = 1 mm

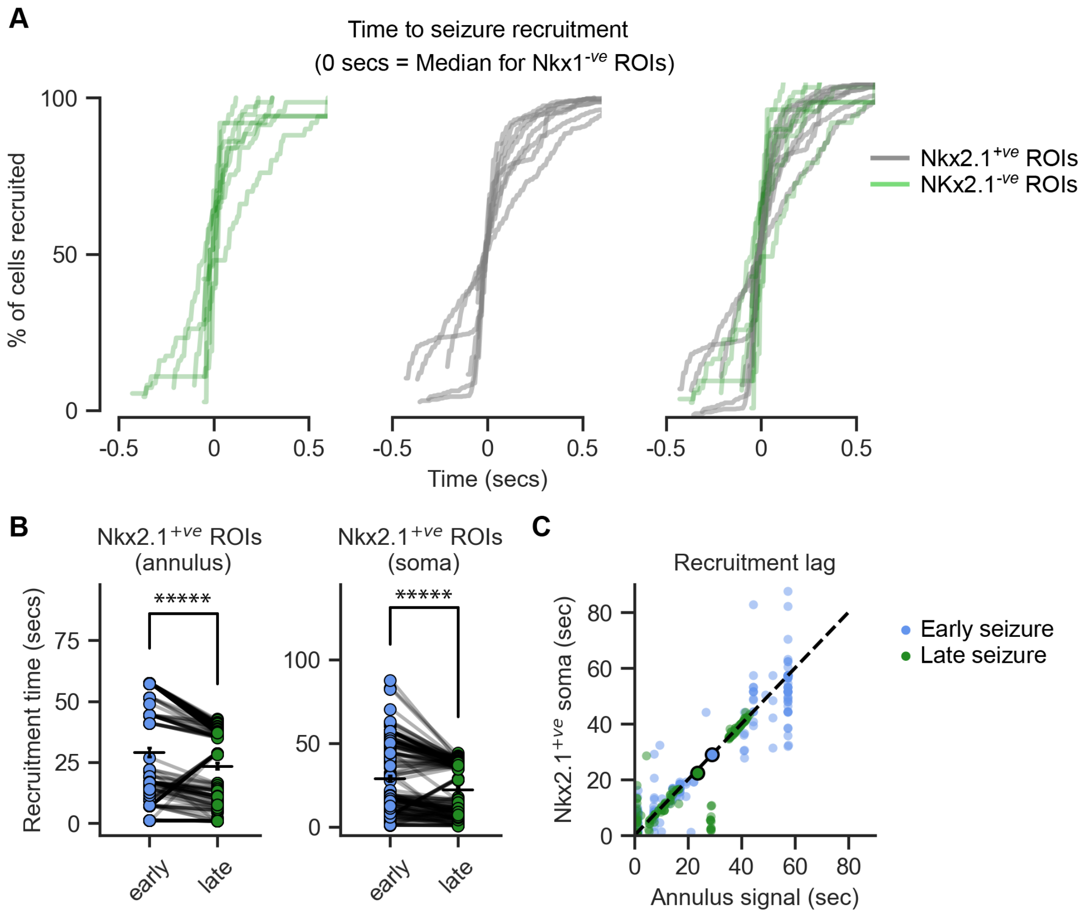

Figure S2. Additional quantification of inhibitory neuron recruitment during seizure propagation. Related to Figure 1.

A. Cumulative time to recruitment of Nkx2.1-expressing inhibitory neurons and Nkx2.1-negative putative-excitatory neurons of all analyzed seizures. Time to recruitment of both Nkx2.1-positive and Nkx2.1-negative cells is normalized to the maximum time to recruitment of Nkx2.1-negative cells of each seizure. Time = 0 sec represents the median time to recruitment of Nkx2.1-negative cells of each seizure.

B. Seizure recruitment time for the annulus (left) and soma (right) of Nkx-2.1-positive ROIs, divided by early and late seizures (from n = 3 mice). The seizure recruitment time is modestly decreased for later seizures for both soma and annulus signals. *****p<10e-5, paired t-test (individual ROIs are compared early vs. late seizures).

C. The recruitment lag of each Nkx2.1-positive ROI's annulus and soma signals, grouped by early (blue) and late (green) seizures. The larger points with black outline represent the mean for each group.

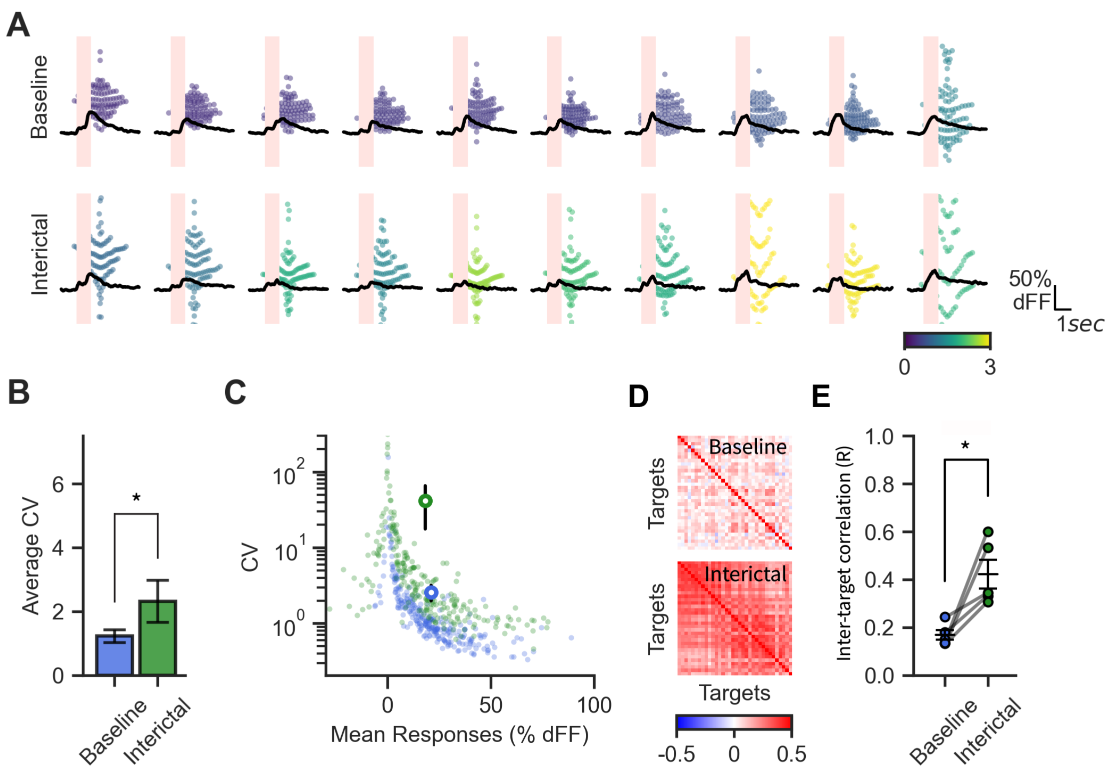

Figure S3. Dynamic variability of neuronal excitability across brain states. Related to Figure 3.

A. The coefficient of variation (CV) measured trial-by-trial variability in photostimulation response magnitude of individual photostimulation targeted neurons. Representative examples of 10 randomly selected individual targets (matched Baseline (top) and Interictal (bottom) states) from a single experiment sorted by increasing CV; black trace is the mean photostimulation timed response, scatter dots are individual photostimulation responses colored by the CV of responses of each respective neuron.

B. Average |coefficient of variation| (CV) of photostimulation targets under baseline and interictal conditions (N = 6 mice; *MWU: $p < 0.05$).

C. Relationship of average photostimulation response magnitude and CV of photostimulation targets across all experiments (2-way ANOVA: p(Binned responses, 20 µm bins) = 3.8e-39, p(Group:Binned responses) = 8.34e-18). Open circles represent mean locations for Baseline (blue circle) and Interictal (green circle) points (errobars: +/- SEM). Excluded datapoints: CV > 300, Mean Responses (% dFF) < -30.

D. Inter-neuronal cross-correlation matrix across all photostimulation targets' z-scored responses to all trials from one representative experiment (left: Baseline, right: Interictal).

E. Increased inter-neuronal correlation of photostimulation targets during interictal period (N=5, paired t-test: *p(baseline vs. interictal)<0.05).

CV: Absolute value of coefficient of variation. Error bars: Mean +/- SEM

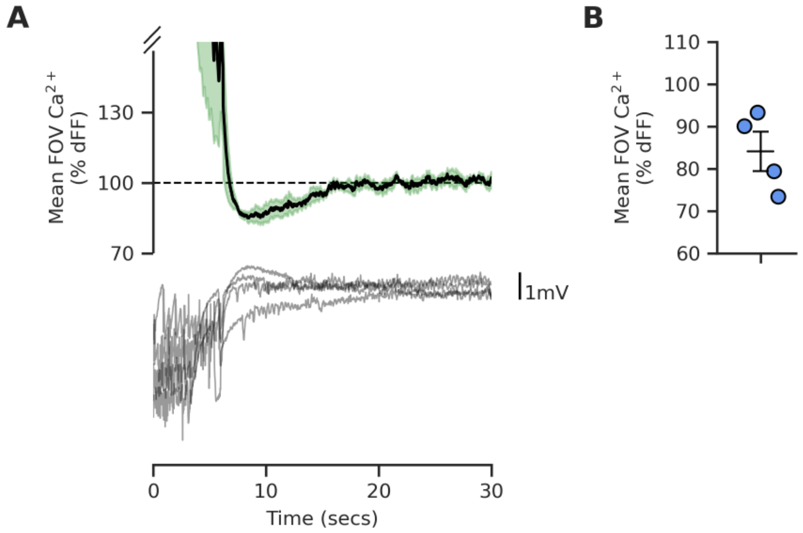

Figure S4. Ca^{2+} signal after seizure termination. Related to Figure 3.

A. Dynamics of the raw FOV Ca^{2+} signal in the post-seizure termination period from a representative experiment (mean +/- SEM; normalised to the interictal period of same experiment).

B. Mean (+/- SEM) of post-seizure termination normalised Ca^{2+} signal across experiments.

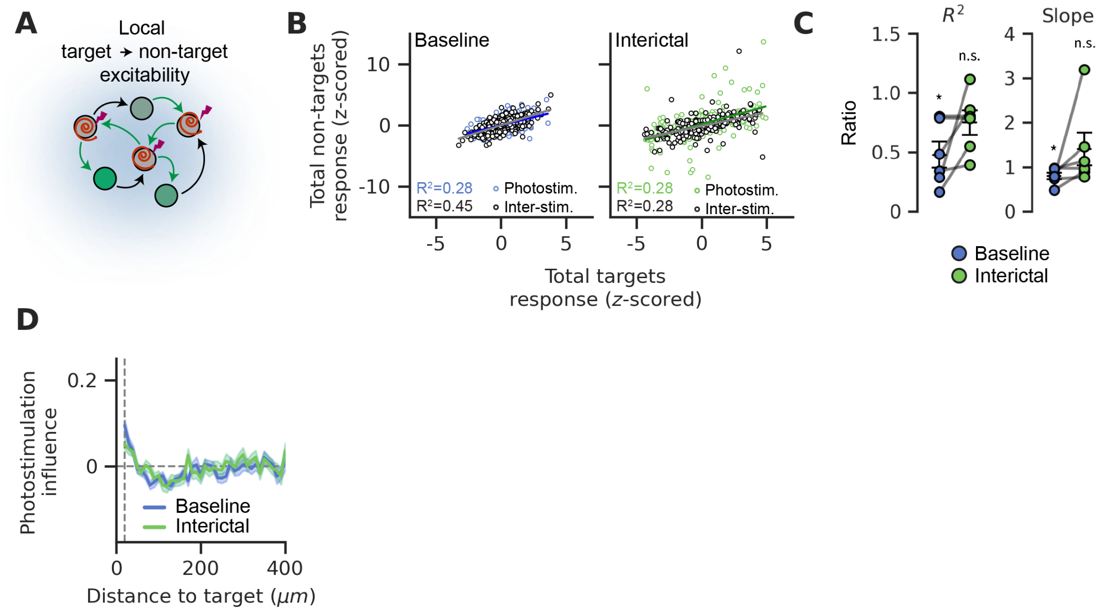

Figure S5. Local circuit excitability in baseline and interictal states. Related to Figure 3.

A. Local circuit excitability is analyzed as the interaction between photostimulation of target neurons and the responses of non-target neurons within the entire FOV of each experiment. The response of each non-target neuron was compared to the response of the closest photostimulation target by Euclidean distance was measured.

B. Relationship of single-trial total response of targeted neurons vs. total response of non-targeted neurons in baseline (left, blue) and interictal (right, green) states. Activity levels of each trial were z-scored within each experiment to allow for comparison across experiments. Inter-stimulation (artificial) trials (open circles) were defined between photostimulation trials to serve as control comparison within each experiment. Each point is a stimulation trial, all datapoints across all experiments are shown (N=6 mice, n = 530 baseline trials and 309 interictal trials, Pearson's R total response targets vs. non targets: p(baseline, photostimulation) = 2.1e-39, p(baseline, inter-stimulation) = 1e-70; p(interictal, photostimulation) = 7.0e-24, p(interictal, inter-stimulation) = 2e-22).

C. R2 and linear regression slope of photostimulation trials vs. inter-stimulation (artificial) trials for baseline (blue) and interictal (green) states (N = 6 mice, Wilcoxon signed-rank test, two-tailed comparison for different than 1, *p < 0.05). The ratio of photostimulation trials to inter-stimulation (artificial) trials is shown to demonstrate that during the baseline state, photostimulation results in lower non-target responses compared to inter-stimulation trials. However, this is not the case in the interictal state, suggesting an increase of local circuit excitability in this state.

D. The influence of photostimulation on the activity of non-target neurons (n = 8567) relative to their distance (10 µm bins) from the nearest target neuron un-

der baseline (blue) and interictal (green) states (2-way ANOVA: p(Distance) = 5.5e-51, p(Group) = 0.80, and p(Group:Distance) = 0.35).

n.s.: not significant. Error bars and spans: Mean +/- SEM.

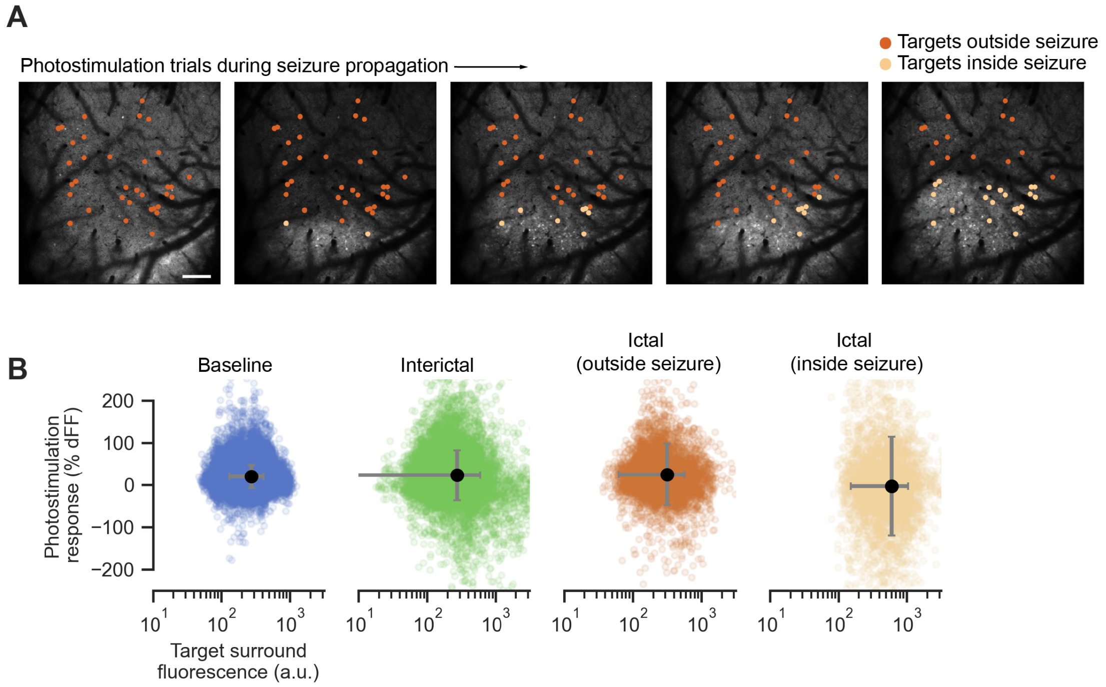

Figure S6: Seizure boundary classification. Related to Figure 4.

A. Calcium fluorescence images of consecutive photostimulation trials during seizure propagation of a single representative ictal event from one experiment. Fluorescence images are 3 secs average around time of photostimulation trial. All photostimulation targets in each experiment were individually classified as being inside or outside the seizure wavefront boundary on a given photostimulation trial during ictal events. Photostimulation trials with unclear seizure wavefront locations (i.e. wavefront has not yet reached FOV, or wavefront has passed FOV) did not receive this classification for targets' location. Scale bar = 200 µm

B. Relationship of surrounding fluorescence (raw) signal to photostimulation response of all targets and photostimulation trials across all experiments. Ictal photostimulation trials were further split by individual targets' location inside/outside the seizure wavefront boundary at each individual photostimulation trial.

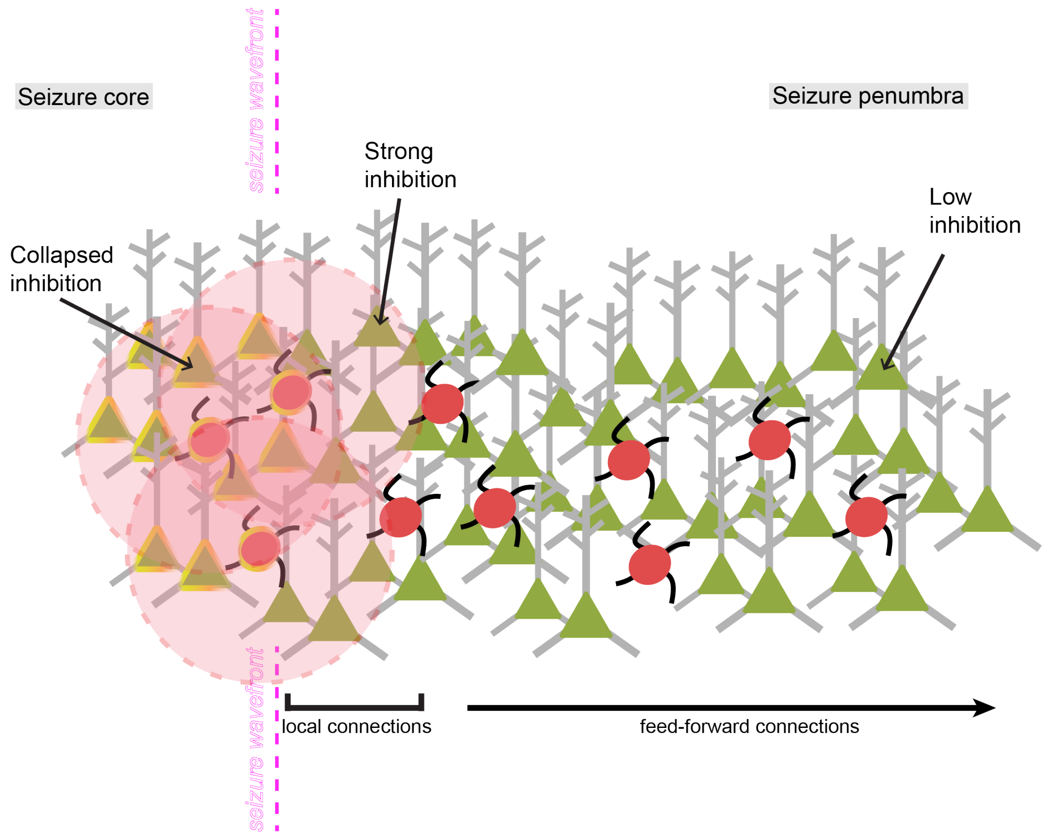

Figure S7: The proposed model of inhibition relative to the seizure wavefront. Related to Figure 4.

The boundary of an actively propagating seizure wavefront includes intense activation of both excitatory (green triangles) and inhibitory neurons (red circles). The intense activation of inhibitory neurons at the edge of the seizure boundary creates a region of strong inhibition in region directly proximal to the seizure wavefront. This occurs in a profile that follows the highly-local anatomical connectivity of cortical interneurons. In contrast, inhibitory neurons in the distal seizure penumbra are not activated ahead of the ictal wavefront, creating a low inhibition state in the distal seizure penumbra.
